# Distinct plastid fructose bisphosphate aldolases function in photosynthetic and non-photosynthetic metabolism in Arabidopsis

**DOI:** 10.1093/jxb/erab099

**Published:** 2021-03-04

**Authors:** Dániel Árpád Carrera, Gavin M George, Michaela Fischer-Stettler, Florian Galbier, Simona Eicke, Elisabeth Truernit, Sebastian Streb, Samuel C Zeeman

**Affiliations:** 1 Department of Biology, ETH Zurich, 8092 Zurich, Switzerland; 5 University of Essex, UK

**Keywords:** Amino acid metabolism, *Arabidopsis thaliana*, Calvin–Benson cycle, fructose-1, 6-bisphosphate aldolase, glycolysis, phloem transport, photosynthesis, plastid metabolism

## Abstract

Plastid metabolism is critical in both photoautotrophic and heterotrophic plant cells. In chloroplasts, fructose-1,6-bisphosphate aldolase (FBA) catalyses the formation of both fructose 1,6-bisphosphate and sedoheptulose 1,7-bisphosphate within the Calvin–Benson cycle. Three Arabidopsis genes, *AtFBA1*–*AtFBA3*, encode plastidial isoforms of FBA, but the contribution of each isoform is unknown. Phylogenetic analysis indicates that *FBA1* and *FBA2* derive from a recently duplicated gene, while *FBA3* is a more ancient paralog. *fba1* mutants are phenotypically indistinguishable from the wild type, while both *fba2* and *fba3* have reduced growth. We show that FBA2 is the major isoform in leaves, contributing most of the measurable activity. Partial redundancy with FBA1 allows both single mutants to survive, but combining both mutations is lethal, indicating a block of photoautotrophy. In contrast, *FBA3* is expressed predominantly in heterotrophic tissues, especially the leaf and root vasculature, but not in the leaf mesophyll. We show that the loss of FBA3 affects plastidial glycolytic metabolism of the root, potentially limiting the biosynthesis of essential compounds such as amino acids. However, grafting experiments suggest that *fba3* is dysfunctional in leaf phloem transport, and we suggest that a block in photoassimilate export from leaves causes the buildup of high carbohydrate concentrations and retarded growth.

## Teaser

Two plastidial aldolases expressed in photosynthetic tissues are crucial for the Calvin–Benson cycle, while a third expressed in heterotrophic tissues is important for glycolysis, amino acid biosynthesis, and photoassimilate translocation.

## Introduction

The fixation of atmospheric CO_2_ into organic compounds by plants occurs via the Calvin–Benson cycle (Calvin, 1962). The cycle is organized into three phases: a CO_2_ fixation phase, a reduction phase and a regeneration phase. Fixation of CO_2_ is catalysed by ribulose-1,5-bisphosphate carboxylase/oxygenase (Rubisco), yielding 3-phosphoglycerate (3PGA). The subsequent actions of phosphoglycerate kinase and glyceraldehyde-3-phosphate dehydrogenase convert 3PGA into triose phosphate (glyceraldehyde 3-phosphate (GAP), which is kept in equilibrium with dihydroxyacetone phosphate (DHAP) by triose phosphate isomerase). At steady state, five out of six triose-phosphates produced remain within the cycle for regeneration of the ribulose 1,5-bisphosphate for CO_2_ fixation, while one can be used to supply substrates for other metabolic processes.

Several major pathways, such as sugar and starch production (Stitt and Zeeman, 2012) and the shikimate pathway (Henkes *et al.*, 2001; Maeda and Dudareva, 2012), draw directly upon Calvin–Benson cycle intermediates. Therefore, the concentrations of intermediates must be carefully managed to meet the demands of these pathways, while maintaining sufficient carbon for RuBP generation. Consequently, the Calvin–Benson cycle and associated pathways are highly regulated at multiple levels (Gontero *et al.*, 2006; Michelet *et al.*, 2013). Sugars derived from the Calvin–Benson cycle can be used locally as building blocks for leaf growth, but a large fraction is transported to heterotrophic sink tissues and catabolized there for energy generation and growth.

The chloroplastic fructose-1,6-bisphosphate aldolase (FBA) takes part in the regeneration phase of the Calvin–Benson cycle (Calvin, 1962) where it performs two specific reactions: (i) the generation of fructose 1,6-bisphosphate (FruBP) from GAP and DHAP and (ii) the production of a sedoheptulose 1,7-bisphosphate (SedBP) from erythrose 4-phosphate (Ery4P) and DHAP. This dual function of the plastidial isoform was shown *in vitro* with enzymatic assays (Brooks and Criddle, 1966; Murphy and Walker, 1981; Flechner *et al.*, 1999) and experimentally using ^14^C-radiolabelled metabolites (Hough and Jones, 1953). Subsequently, FruBP and SedBP are dephosphorylated by fructose-1,6-bisphosphatase (FBPase) and sedoheptulose-1,7-bisphosphatase (SBPase), respectively (Tamoi *et al.*, 2006; Liu *et al.*, 2012).

There are multiple *FBA* genes annotated in higher plant genomes, with the exact number varying amongst species (Cai *et al.*, 2016; Lv *et al.*, 2017). In Arabidopsis there are eight genes, with three predicted to encode plastid-localized isoforms and five to encode cytosolic isoforms (Lu *et al.*, 2012). FBA1 and FBA2 were shown to be localized to the plastid using fluorescent fusion proteins in Arabidopsis leaf mesophyll protoplasts (Vidi *et al.*, 2006), while FBA3 was found in chloroplast preparations using proteomic approaches (Ytterberg *et al.*, 2006). Five further FBAs (FBA4–FBA8) are predicted to be localized in the cytosol (Lu *et al.*, 2012; Cai *et al.*, 2016), and this localization for FBA8 was confirmed experimentally with fluorescent fusion proteins (Garagounis *et al.*, 2017). In the cytosol, FBA also catalyses the reversible condensation of triose phosphates to FruBP (Lebherz *et al.*, 1984). Cytosolic FBA mutants have not yet been fully characterized, although *fba8* mutants showed a significant reduction in the total FBA enzyme activity in roots and a reduced growth rate (Garagounis *et al.*, 2017).

Silencing of a gene encoding a plastidial FBA (pFBA) with an antisense approach led to a decrease in photosynthesis, starch synthesis, activity of other Calvin–Benson cycle enzymes, and plant growth in potato (Haake *et al.*, 1998). The knock-down of the targeted pFBA protein was confirmed on a transcriptional and translational level, but the isoform specificity was not determined and a simultaneous impact on the cytosolic FBAs may have occurred. On the other hand, overexpression of pFBA in tobacco led to an enhanced photosynthetic rate and increased biomass at elevated CO_2_ levels (Uematsu *et al.*, 2012). The capacity to control the flux through the Calvin–Benson cycle was exploited in transgenic tobacco and Arabidopsis plants (Simkin *et al.*, 2015, 2016), where pFBA was overexpressed in combination with other enzymes of photosynthetic metabolism (SBPase and glycine decarboxylase-H protein). This resulted in an increase in carbon assimilation and total biomass compared with wild-type plants. Increased pFBA activity was crucial to obtain maximum yield but was insufficient alone, at least at ambient CO_2_ levels (Simkin *et al.*, 2015, 2016). Despite this impressive research and evidently important role for pFBA, surprisingly little is actually known about the individual isoforms. The Arabidopsis *fba2* mutant was shown with qualitative iodine staining to have decreased starch levels, but quantitative measurements were not presented (Lu *et al.*, 2012). An *in vitro* experiment measured the activity of recombinant FBA2 and FBA3 proteins from Arabidopsis, which displayed similar kinetic properties (Mininno *et al.*, 2012).

Multiple pFBA isoforms might imply genetic redundancy, a diversification of function, or a spatio-temporal separation of activities. Here, we aimed to resolve the roles of the individual pFBA isoforms. Using a series of loss-of-function mutants, we show which isoforms are required for Calvin–Benson cycle function, and to what extent. We also show that FBA3, rather than functioning in photosynthetic CO_2_ assimilation, is involved in plastid glycolytic metabolism in heterotrophic tissues, and suggest that this is crucial for phloem function.

## Materials and methods

### Phylogenetic and bioinformatics analysis

The amino acid sequences of the proteins for phylogenetic analyses were acquired from the National Center for Biotechnology Information and Phytozome databases using pBLAST against each of the three plastidial FBA (NP_565508.1, NP_568049.1, NP_178224.1) sequences from *A. thaliana*. The alignment was assembled with MAFFT and used to calculate sequence similarity (Katoh and Standley, 2013). The corrected Akaike information criterion on the ProtTest Server (Abascal *et al.*, 2005) was used to choose the best suitable model (JTT) for building the tree. The final trees were constructed with Mega6 (Tamura *et al.*, 2013) and branch support was tested by running 1000 bootstrap replicates. The gene duplication of the pFBAs in the Arabidopsis genome was checked by using the Plant Genome Duplication Database (Lee *et al.*, 2013). Peptides and transcripts detected in publicly available experiments were checked using the pep2pro (Baerenfaller *et al.*, 2011), Genevestigator, and eFP Browser tools (Zimmermann *et al.*, 2004; Winter *et al.*, 2007).

### Plant material and growth

Arabidopsis plants (Col-0 and L*er* ecotypes) were grown in Percival AR95 chambers (CLF Plant Climatics) on a nutrient-rich, medium-grade, peat-based soil or on agar plates supplied with half-strength Murashige and Skoog (MS) medium (Duchefa Biochemie) at constant 20 °C, 70% relative humidity, with a 12 h light–12 h dark diel cycle. Uniform light intensity was set to 150 μmol m^−2^ s^−1^. Sown seeds were stratified for 48 h at 4 °C. Single, homozygous T-DNA insertion mutants were isolated: *fba1*-1 (SALK_063223; Col-0), *fba1*-2 (SAIL_752_G05C; Col-0), *fba2*-1 (SALK_000898; Col-0), *fba2*-2 (SALK_073444; Col-0), *fba3*-1 (GT12795.Ds5; L*er*), *fba3*-2 (SALK_092715; Col-0). T-DNA insertion-specific and gene-specific primers (see Supplementary Table S1) were used for genotyping. To create a double mutant, *fba1*-1 and *fba2*-1 were crossed. The *fba3* mutant and its wild type were also grown on plates with additional sucrose (0.1, 0.5, 1, and 1.5%) for root length measurements. Grafting of wild-type and *fba3* mutant seedlings was performed according to Melnyk (2017). Rosettes for growth measurement were photographed every third day 12–28 d after germination to quantify leaf area. Pictures were analysed using ImageJ software.

### Creation of expression reporter constructs and Arabidopsis transformation

The *FBA* promoter (2000 bp upstream of the starting ATG codon) sequences were amplified from genomic DNA using iProof polymerase (Bio-Rad). Promoters were cloned into the entry vector pDONR P4-P1r. The β-glucuronidase (GUS) coding sequence was cloned into pENTR/SD/D-TOPO vector (Thermo Fisher Scientific). The promoters were placed upstream of the GUS coding sequence in the destination vector pB7m24GW using Multisite Gateway cloning. The resultant promoter–GUS constructs were introduced into wild-type Arabidopsis via *Agrobacterium tumefaciens*-mediated transformation (Clough and Bent, 1998). Selection of transformants with BASTA was performed on soil-grown seedlings.

### Promoter activity analysis of the pFBAs using GUS assay

Arabidopsis seedlings expressing promoter–GUS reporter constructs were grown on half-strength MS agar plates or on soil. Plant material was destained in 90% acetone and incubated in staining solution (0.1 M phosphate buffer, 0.2% (v/v) Triton X-100, 2 mM potassium ferrocyanide, and 2 mM potassium ferricyanide) with 2 mM X-GLUC in *N*,*N*-dimethlyl formamide at 37 °C. Samples were dehydrated with an ethanol series (20, 35, 50%), fixed with 50% ethanol, 3.7% (v/v) formaldehyde, 5% (v/v) acetic acid, and kept in 70% ethanol. Root samples were embedded in resin (Kalve *et al.*, 2015) and sectioned (80 µm) on a rotary microtome (Leica RM 2155). Leaf sections were made by hand. Plants and sections were visualized by light microscopy (Imager Z2 (Carl Zeiss) and VHX 1000D (Keyence)). At least two independent transformed lines were analysed for each reporter construct.

### Quantitative reverse transcription PCR measurements

*pFBA* gene expression was measured with RT-qPCR. Primers were preferentially designed at exon–intron borders to avoid gDNA contamination (see Supplementary Table S1). Total RNA was isolated with Isol-RNA Lysis Reagent (5 PRIME); cDNA synthesis was carried out with SuperScript III first strand kit (Thermo Fisher Scientific) applying an oligo(dT) primer. The PCR reaction was performed with Fast SYBR Green Master Mix (Thermo Fisher Scientific) on a 7500 Fast Real-Time PCR system (Applied Biosystems). Expression of each *FBA* was normalized to the *YSL8*, *ACTIN2*, and *GAPC2* reference genes. The wild-type expression for each gene was set to 1 for comparisons with knock-out mutants.

### Total FBA enzyme activity measurement

Whole rosettes from soil-grown plants or roots from plate-grown plants were homogenized in extraction medium (50 mM HEPES–KOH, pH 7.5, 10 mM MgCl_2_, 1 mM EDTA, 0.25% (w/v) BSA; 100 mg plant material per ml extraction medium) to extract total soluble proteins. After clarification by centrifugation (10 000 *g*, 10 min, 4 °C), the extract was further diluted with extraction medium. The cyclic assay for FBA activity was performed using an adaptation of previously reported methods (Haake *et al.*, 1998; Gibon *et al.*, 2004). FBA was assayed using a stopped assay, in which aliquots of extracts are incubated with FruBP for a fixed time with coupling enzymes that convert the products to glycerol 3-phosphate. After stopping the reaction, the product amount was then determined in a cycling assay, wherein the net rate of the cycle depends on the initial glycerol 3-phosphate concentration (Gibon *et al.*, 2004). Briefly, the first assay mixture consisted of 100 mM Tricine–KOH, pH 8, 5 mM MgCl_2_, 1 mM EDTA and 0.05% (v/v) Triton X-100, 1 unit ml^−1^ triose-phosphate isomerase, 2 units ml^−1^ glycerol-3-phosphate dehydrogenase, 5 mM FruBP, and 0.3 mM NADH. After adding protein extract, the mixture was incubated for 20 min at 25 °C. Replicate assays without FruBP served as controls. Reactions were stopped by acidification with HCl. After neutralization, glycerol 3-phosphate was measured by a glycerol-3-phosphate dehydrogenase/glycerol-3-phosphate oxidase cycling assay. The second assay mixture contained 100 mM Tricine–KOH, pH 8, 2 mM MgCl_2_, 0.5 units ml^−1^ triose-phosphate isomerase, 1 unit ml^−1^ glycerol-3-phosphate dehydrogenase, 2.5 units ml^−1^ glycerol-3-phosphate oxidase, 0.6 mM NADH. The assay mixture was incubated at 25 °C and absorbance at 340 nm monitored to determine the rate of NADH depletion.

### Starch and metabolite measurements

Samples comprising all the leaves of individual rosettes were frozen in liquid N_2_ and pulverized frozen using a mixer mill (Retsch MM 301). For chlorophyll determination, 34-day-old plant material was extracted in 80% aqueous acetone for 1 h on ice in the dark. After centrifugation (14 000 *g*, 2 min, 20 °C), the absorbance at 645 nm and 663 nm was determined and total chlorophyll content calculated according to Arnon (1949). For other metabolites, plant material was extracted according to Arrivault *et al.* (2009), with modifications. Briefly, 15 mg of plant powder was mixed with 300 µl chloroform:methanol solution (3:7 (v/v)). The mixture was incubated at −20 °C for 2 h. Cold water (400 µl) was added, the sample mixed and the phases separated by centrifugation (10 000 *g*, 10 min, 4 °C). The aqueous phase was collected, dried under vacuum and stored at −80 °C. For starch measurements, the starch-containing insoluble pellet was washed once with 70% ethanol and then with water before being stored at −20 °C.

Starch and sugars were measured by modifying earlier protocols (Egli et al., 2010). The fraction containing soluble sugars (glucose, fructose, and sucrose) was applied to sequential 1.5 ml columns of Dowex-50W and Dowex-1 (Sigma-Aldrich). Neutral compounds were eluted with 5 ml of water, lyophilized, and dissolved in 200 μl of water. Cellobiose (5 nmol per 5 mg plant material) was added as an internal standard. Sugars were separated by anion exchange chromatography (CarboPac PA-20 column) and detected with pulsed amperometric detection (HPAEC-PAD, Thermo Fisher Scientific). The eluents and gradients are described in Supplementary Table S2. Peaks were quantified using Chromeleon software (Thermo Fisher Scientific) against standard curves of pure compounds and normalized to the internal standard. Starch in the insoluble fraction was measured as described previously (Smith and Zeeman, 2006).

For UHPLC-MS/MS measurements, samples were dissolved in water (250 µl/15 mg plant material) and filtered (Minisart RC4, pore size: 0.2 µm, Huberlab). Metabolites were separated by UHPLC (Agilent 1290 Infinity, Agilent Technologies, USA) with an Acquity HSS T3 C18 end-capped reverse phase column (100 Å pore size, 1.8 µm particle size, 2.1×150 mm; Waters Corp., USA) coupled to a QTRAP 5500 triple quadrupole MS (AB Sciex). Ion-pair reversed-phase chromatography was performed according to Büescher *et al.* (2010), using eluents and gradients described in Supplementary Table S3. Eluted compounds were automatically quantified against standard curves of pure compounds using the software Multiquant (AB Sciex, Switzerland). When necessary, plant extracts were spiked with pure components to confirm the peak identity. Peaks for ADP, ATP, FruBP, GAP, isocitrate, 3PGA, phosphoenolpyruvate (PEP), pyruvate, RuBP, ribulose 5-phosphate, threonine, and xylulose 5-phosphate were manually quantified. Metabolite amounts were normalized to the wild type for heatmap visualization. Principal component analysis (PCA) of the metabolomics data was performed with ClustVis (Metsalu and Vilo, 2015).

### Gas exchange

Gas exchange experiments were carried out using three to four mutant and three to four wild-type plants in parallel, as described by Kölling et al. (2015a). Data were processed using custom-built software described by George *et al.* (2018). Plants were introduced into measurement chambers a day before measurement to allow acclimation, then measured continuously for a full day and night. Plants were subsequently harvested and weighed. CO_2_ assimilation was normalized to fresh weight.

### Microscopy and tissue staining

To visualize leaf vascular patterning, leaves were incubated for 1 h in a 3:1 mixture of 95% ethanol and acetic acid at 20 °C. Leaves were washed twice for 30 min in 70% ethanol, then kept for 16 h in 100% ethanol at 4 °C. After clearing in 10% (w/v) NaOH for 1 h at 37 °C, leaves were mounted on glass slides in 50% (v/v) glycerol and imaged by dark-field microscopy (Leica M205 microscope).

## Results

### Conservation and duplication of plastidial FBAs in the plant kingdom

**Fig. 1.**
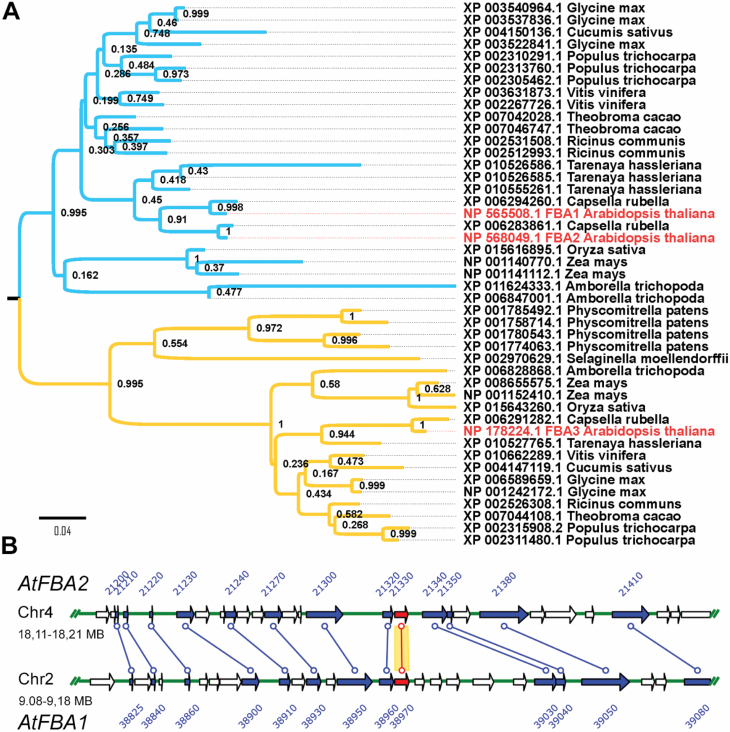
Phylogenetic analysis of plastidial FBAs. (A) Maximum likelihood phylogenetic tree of the plastidial FBA proteins from mosses and higher plant species, with Bootstrap values. The Arabidopsis proteins (NP_565508.1, NP_568049.1, NP_178224.1) are shown in red. (B) Duplicated chromosomal regions of the Arabidopsis genome contain the *FBA1* and *FBA2* genes according to the Plant Genome Duplication Database. Arrows represent genes on distinct, 100 kbp regions of chromosomes 2 and 4. Lines connect duplicated genes including *FBA1* and *FBA2* (in red).

Previous phylogenetic analyses of the FBA protein sequences have revealed that plants contain multiple FBA-encoding genes that divide into two subfamilies respectively containing cytosolic and plastid-targeted isoforms (Lu *et al.*, 2012). Arabidopsis has three plastidial and five cytosolic isoforms. We analysed the plastidial isoforms further; the similarity of the Arabidopsis protein sequences was high (above 80%) with FBA1 (At2g21330) and FBA2 (At4g38970), being especially similar (95%; see Supplementary Table S4). FBA3 (At2g01140) was less similar and phylogenetic analyses revealed that it clustered with the FBAs found in both angiosperms and more primitive plant species such as *Physcomitrella patens* and *Selaginella moellendorffii* (Fig. 1A). AtFBA1 and AtFBA2 clustered with FBAs found only in angiosperms (Fig. 1A), including *Amborella trichopoda* (considered as a basal angiosperm; Albert *et al.*, 2013). Gene duplication of plastidial FBAs in both clusters was common. A duplication event within the *Brassicaceae* is responsible for *FBA1* and *FBA2* genes. In Arabidopsis, *FBA1* and *FBA2* are located on chromosome II and IV, respectively, within syntenic zones (Fig. 1B) approximately 0.4 Mb in length containing similar sets of 56 genes.

### Plastidial FBAs display two distinct expression patterns

**Fig. 2.**
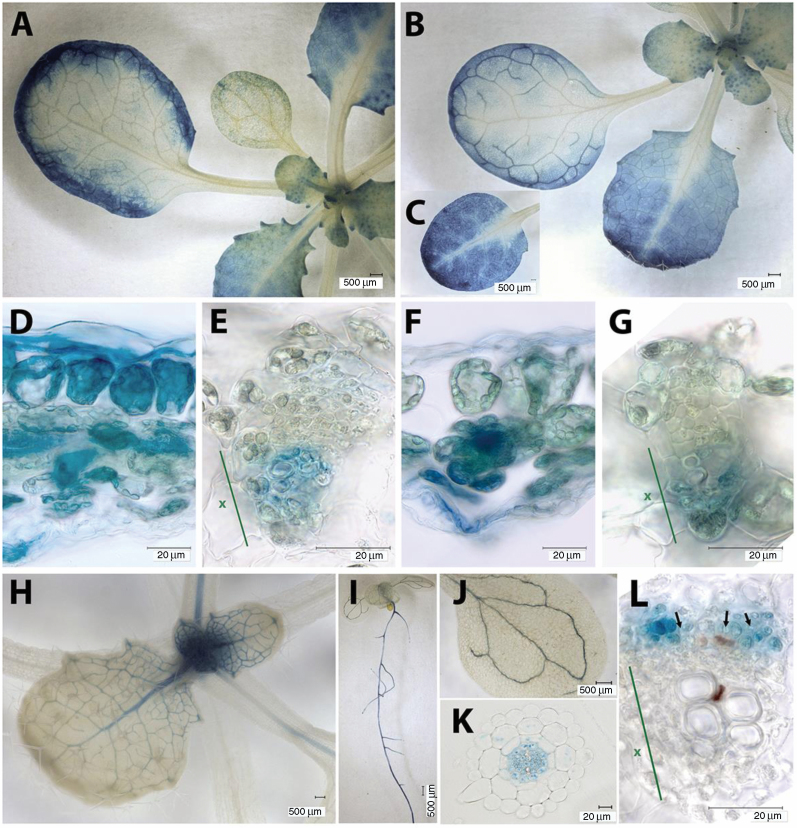
Expression patterns of *FBA1*, *FBA2*, and *FBA3* genes in Arabidopsis. Staining of plants transformed with *FBA1*_*pro*_*::GUS* (A, D, E), *FBA2*_*pro*_*::GUS* (B, C, F, G), and *FBA3*_*pro*_*::GUS* (H–L) constructs in developing leaves (A, B, C, H), sections through leaf margins (D, F), leaf vascular bundles (E, G, L), the whole seedling (I), cotyledon (E), and root cross-section (K). Representative images of expression patterns observed in at least two independent transformants are shown. In (E, F, L), the position of the xylem is indicated (x). In (L), some sieve tube cells are indicated (black arrows).

**Fig. 3.**
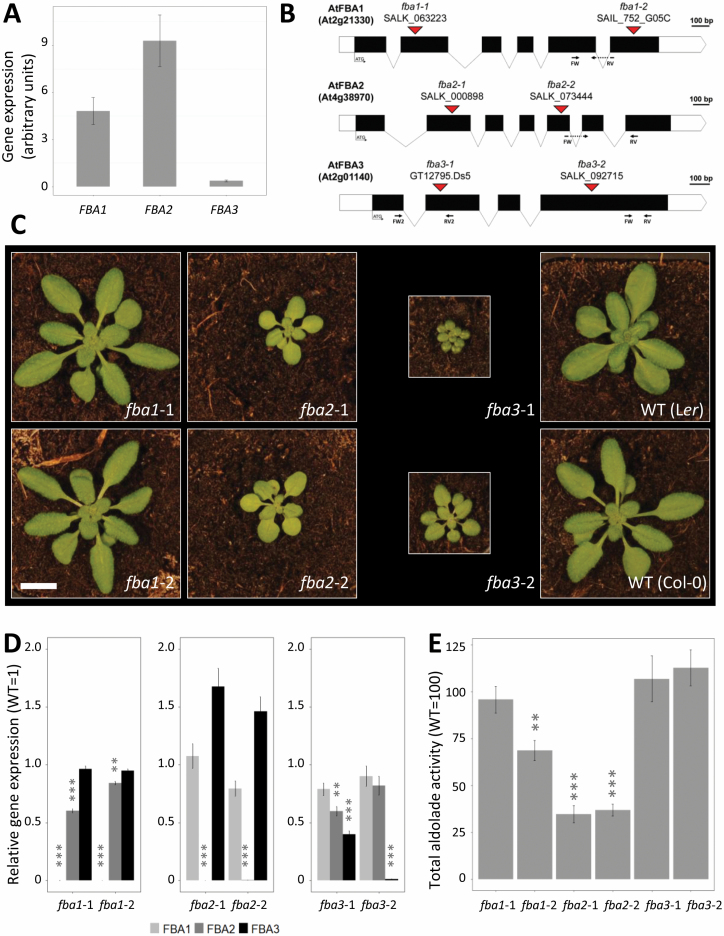
Homozygous knockout mutants of the three plastidial FBA isoforms. (A) Expression of the plastidial *FBA* genes in the shoot of wild-type Col-0 plants measured with RT-qPCR at midday. Values are normalized to the housekeeping genes *AtYLS8*, *ACTIN2*, and *GAPC2*, and are means of three biological replicates (±SE). (B) Structure of the plastidial *FBA* genes: black and white rectangles represent the exons and the UTR regions respectively, with introns represented as lines. The locations of the T-DNA insertions in the different mutant alleles are indicated by red triangles. Annealing sites of primers used for RT-qPCR are indicated. (C) Phenotype of the mutants 30 d after germination. Scale bar: 1 cm. (D) Expression of the *FBA1*, *FBA2*, and *FBA3* genes in the rosettes of the single knock-out mutants. Values are normalized to *AtYLS8*, *ACTIN2*, and *GAPC2*, and are means of three biological replicates (±SE), and are expressed relative to the mean value for the wild type. (E) Total FBA enzyme activity measured in the shoots of the *fba1*, *fba2*, and *fba3* mutants. The enzyme activity is normalized to the respective wild type. Values are means of three biological replicates (±SE). **P≤0.05, ***P≤0.01: significant differences from the corresponding wild type. The mean activity (±SE) of Col-0 wild type from three experimental replicates was 4.41 ± 1.00 μmol min−1 g−1 fresh weight.

We analysed the expression patterns of the three pFBA-encoding genes in Arabidopsis using publicly available microarray datasets and by analysing transformed plants harboring promoter–GUS fusion constructs. Expression patterns visualized using the eFP Browser (Winter *et al.*, 2007) revealed that *FBA1* and *FBA2* were both expressed primarily in green tissues (e.g. cotyledons, rosette and cauline leaves, stems, sepals, and developing siliques). Little or no expression was detected in non-green tissues (e.g. roots, sepals, anthers, and pollen) or later in seed development (see Supplementary Fig. S1A, B). In contrast, *FBA3* expression was relatively high in most non-green tissues, and lower in green tissues (Supplementary Fig. S1A, B). These expression patterns broadly agree with previous studies (Lu *et al.*, 2012), with the quantification of FBA specific peptides in tissue specific protein samples (Supplementary Fig. S1C), and with similar gene expression analyses performed with Genevestigator® (Supplementary Fig. S1D). Our analysis of plants expressing promoter–GUS fusion constructs further supported these patterns and provided additional detail (Fig. 2). Plants containing *FBA1*_*pro*_*::GUS* and *FBA2*_*pro*_*::GUS* constructs both showed a strong GUS staining in the leaf lamina, especially in younger leaves and around the margins of older leaves (Fig. 2A–D, F), where *FBA2*_*pro*_*::GUS* constructs gave stronger GUS-staining than *FBA1*_*pro*_*::GUS* constructs. In the leaf vasculature, staining was visible in cells adjacent to the xylem for both *FBA1*_*pro*_*::GUS* and *FBA2*_*pro*_*::GUS* constructs (Fig. 2E, G). No root staining was detected for plants transformed with either of these construct, however. The pattern was different in plants containing *FBA3*_*pro*_*::GUS* constructs (Fig. 2H–L), where strong staining was seen in roots, particularly the stele (Fig. 2I, K). In leaves and petioles, expression was restricted to the vasculature, with no detectable staining in the leaf lamina (Fig. 2H, J). Within the leaf vasculature, GUS staining was visible in cells adjacent to the phloem (Fig. 2L). RT-qPCR confirmed that *FBA3* transcripts were detectable in leaves (Fig. 3A), even if at much lower levels than *FBA2* and *FBA1* transcripts.

### Isolation of T-DNA insertion single knock-out mutants

To investigate the roles of the three Arabidopsis pFBA isoforms, two independent homozygous T-DNA insertion lines were isolated for each gene: *fba1*-1, (SALK_063223), *fba1*-2 (SAIL_752_G05C), *fba2*-1 (SALK_000898), *fba2*-2 (SALK_073444), *fba3*-1 (GT12795.Ds5), and *fba3*-2 (SALK_092715). All mutants are in the Col-0 background except for *fba3*-1, which is in the L*er*-0 background. The T-DNA insertions were located in exons in each of the alleles (Fig. 3B). The *fba1* mutants displayed a wild-type growth phenotype, while the *fba2* mutants grew more slowly than the wild type and were significantly smaller (Fig. 3C; Supplementary Table S5). In both genetic backgrounds, *fba3* mutants displayed the most severe phenotype, growing very slowly compared with the other genotypes. The *fba2* mutants appeared paler than the wild type while the *fba3* mutants appeared darker. However, chlorophyll measurements did not reveal any consistent differences from the respective wild types (Supplementary Table S5). In each mutant, we analysed the transcript levels for each of the three genes using RT-qPCR with primers annealing toward the 3′ end (Fig. 3B; Supplementary Table S1). Transcript levels for each mutated gene were very low, except in *fba3*-1, where transcript levels were around 40% that of the wild type, possibly due to an aberrant mRNA produced from within the T-DNA (Fig. 3D). We therefore performed a second RT-qPCR reaction for *fba3*-1 using primers spanning the T-DNA insertion site. In this case, essentially no transcript was detected (less than 0.1% of the wild-type value). In each mutant, the expression of the other two *pFBA* genes was either unaltered or only slightly changed (FBA2 expression was slightly reduced in both *fba1* mutant alleles, for example; Fig. 3D).

FBA catalyses the condensation of DHAP with either GAP or Ery4P, yielding FruBP or SedBP, respectively (referred to as FBA or SBA reactions). Total enzyme activity (measured via the FBA reaction) was determined in crude extracts of the rosettes of each of the mutant alleles (Fig. 3E). Loss of FBA1 led to either no change (in *fba1*-1) or a slight drop (in *fba1*-2) in total activity, while loss of FBA2 resulted in a substantial decrease in activity, with both alleles having only one-third that of the wild type. Loss of FBA3 had no impact on the total activity. This major contribution of FBA2 is consistent with early observations that plastidial isoforms contribute 85–95% of the total FBA activity in leaves (Schnarrenberger and Krüger, 1986), and with the relative expression levels of the three *pFBA* genes (Fig. 3A). The remaining activity in the *fba2* mutant is presumably contributed by the remaining plastidial and cytosolic isoforms. In roots, no significant differences in FBA activity were detected in the three single mutants, even though *FBA3* is expressed in the root (with activities of 101±9% for *fba1*-1, 101±11% for *fba2*-1, and 94±6% for *fba3*-1, compared with their respective wild types; *n*=3, mean ±SEM). This result could signify that the cytosolic isoforms contribute most of the activity in this organ.

### FBA1 and FBA2 are partially redundant and essential for photosynthesis

Considering that FBA1 and FBA2 resulted from a relatively recent gene duplication and share similar expression profiles in green tissues, we considered that there may be functional redundancy between them. Therefore, we crossed *fba1-1* with *fba2-1* and genotyped 268 individuals from the segregating F_2_ population. The observed ratio of genotypes was significantly different from that expected for two unlinked mutations (see Supplementary Table S6). Only one double mutant seedling was found, which was yellow and not viable beyond the cotyledon stage. Fewer than expected plants being homozygous for one mutant and heterozygous for the other were observed (particularly *fba1*^(+/−)^*fba2*^(−/−)^), although these plants were capable of developing rosettes, flowering, and setting seeds. Opening the developing siliques of self-pollinated *fba1*^(−/+)^*fba2*^(−/−)^ or *fba1*^(−/−)^*fba2*^(+/−)^ plants revealed a high proportion of undeveloped or aborted seeds (Supplementary Fig. S2). These observations suggest that plastidial FBA activity conferred by FBA1 and/or FBA2 is required for normal seed development, explaining the distorted segregation and why so few homozygous double mutants were obtained. These data also suggest that, despite being expressed in the developing seeds, FBA3 is insufficient to substitute for FBA1 and FBA2 (Supplementary Fig. S1).

**Fig. 4.**
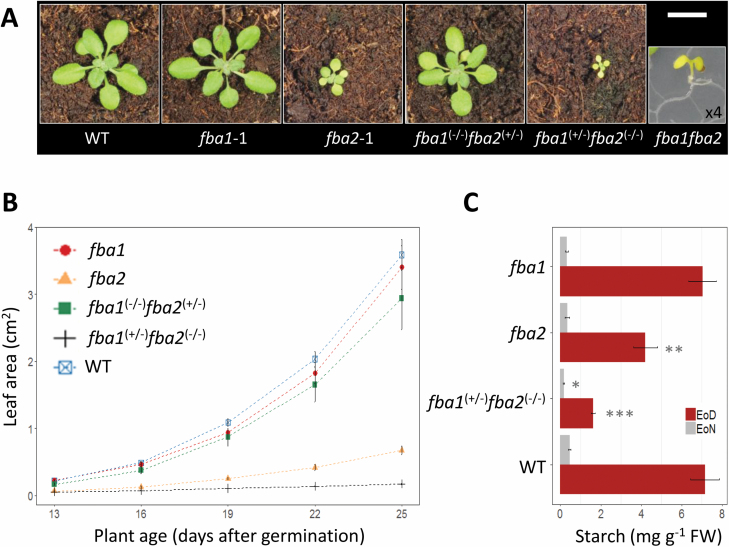
Phenotypes of mutants deficient in both FBA1 and FBA2. (A) Appearance of wild-type, *fba1*, and *fba2* plants and of representative genotypes isolated from the segregating F_2_ population of a cross between *fba1* and *fba2*. All plants were 25 d old and imaged from the growth analysis shown in (B), except the *fba1fba2* double mutant, which was the sole individual identified during segregation analysis and is ×4 magnified (see Supplementary Table S6). Scale bar: 1 cm. (B) Growth rate of the different genotypes in (A) (except for the *fba1fba2* double mutant) from 13–25 d after germination. Values are the means of 6–11 biological replicates (±SE). (C) Starch content of *fba1*, *fba2*, *fba1*^(+/−)^*fba2*^(−/−)^, and of the wild type. Values are the means of four biological replicates (±SE). EoD, end of day; EoN, end of night. *P≤0.1, **P≤0.05, ***P≤0.01: significant differences from the corresponding wild type.

Interestingly, we also observed different FBA gene dosage effects on plant growth; the *fba1*^(+/−)^*fba2*^(−/−)^ plants had a very severe growth phenotype, with much smaller rosettes than the *fba2* single mutant (Fig. 4A, B). In contrast, the *fba1*^(−/−)^*fba2*^(+/−)^ plants maintained a near-wild-type growth phenotype under the conditions used (Fig. 4A, B). Thus, while one functional copy of FBA2 appears sufficient to maintain vegetative growth, having only FBA1 is limiting, and reducing this to a single functional copy limits growth even further. To get an impression of the impact of limiting FBA activity on photosynthetic metabolism in Arabidopsis, we analysed these *fba1* and *fba2* mutants for the accumulation of starch—a primary photosynthetic product that accumulates during the day (Streb and Zeeman, 2012). The *fba1* mutants had similar starch levels to the wild type at the end of the day, while the *fba2* mutants had a significant 40% reduction and the *fba1*^(−/+)^*fba2*^(−/−)^ plants had an 80% reduction (Fig. 4C).

### pFBA mutants have distinct changes in photosynthesis, respiration, and carbohydrate metabolism

**Fig. 5.**
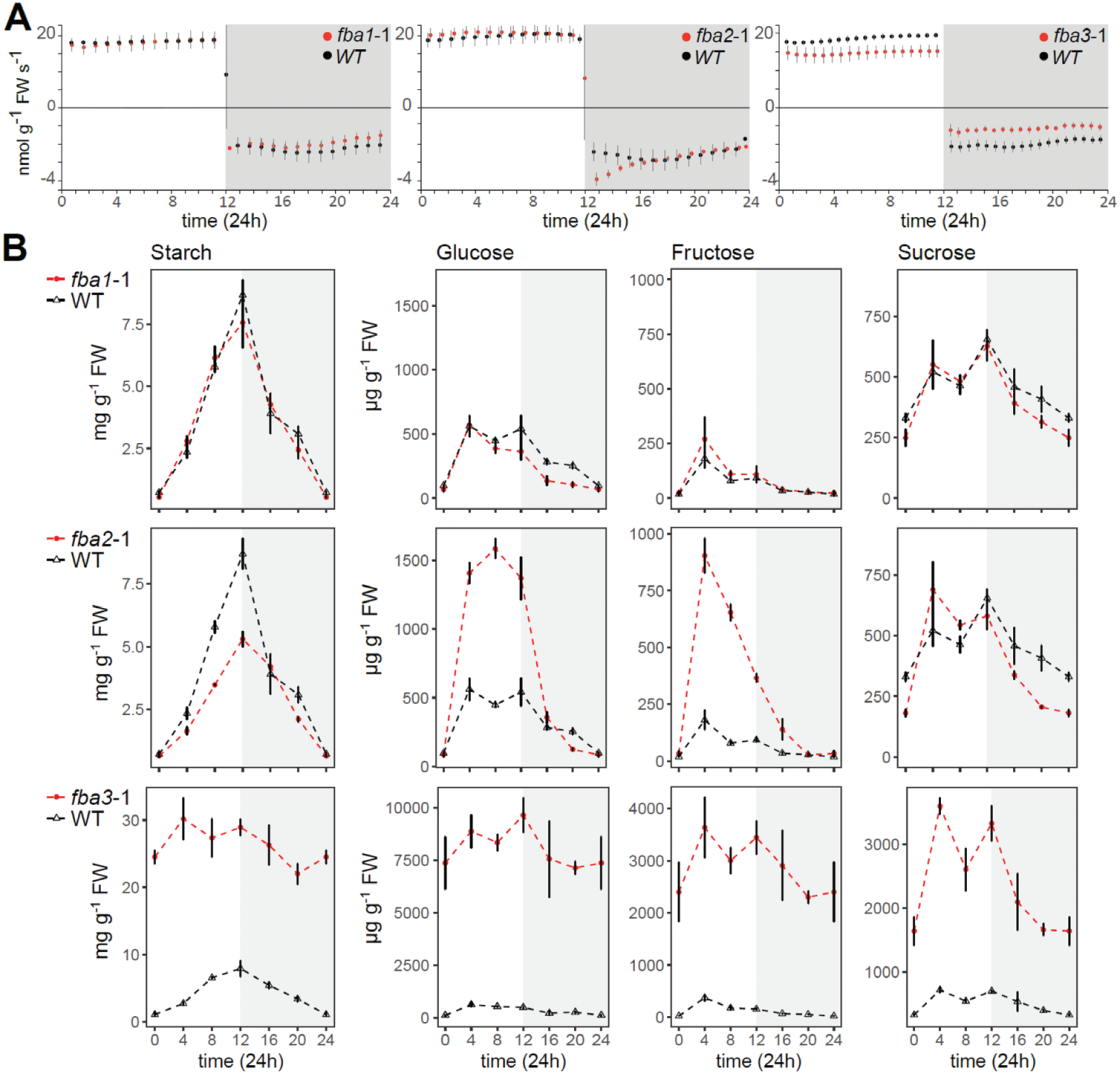
Photosynthesis, respiration, and diel changes in carbohydrate levels in *fba1*, *fba2*, and *fba3* mutants. (A) Daytime photosynthetic carbon assimilation and night-time respiration of the aerial parts of 28-day-old plants (50-day-old for *fba3*), measured by infrared gas analysis (illumination at 150 μmol m^−2^ s^−1^, 20 °C, 70% relative humidity, 380 ppm CO_2_). Note the difference in scale of the *y*-axis between positive and negative values. Mean values from four biological replicates (±SE) are given. A rate of 20 nmol g^−1^ FW s^−1^ for the wild type equates to approximately 5 μmol m^−2^ projected leaf area s^−1^. (B) Starch and the soluble sugars glucose, fructose, and sucrose were measured in the aerial parts of 25-day-old plants over a 12 h day (white background)–12 h night (grey background) cycle. Note the differences in the *y*-axis scale depending on the measured data in the different mutants. The same data for the wild type (in black) are compared against each mutant. Mean values from four biological replicates (±SE) are given.

To investigate photosynthetic CO_2_ assimilation and respiration in FBA-deficient plants in more detail, the single mutants were monitored via infrared gas analysis in custom-made chambers (Fig. 5A; Kölling et al., 2015a; George *et al.* 2018) using the same conditions as for plant cultivation (illumination at 150 μmol m^−2^ s^−1^, 20 °C, 70% relative humidity, 380 ppm CO_2_). The photosynthetic CO_2_ assimilation rates in *fba1* and *fba2* plants were similar to the wild type, which for *fba2* was slightly unexpected considering its slower growth rate (Fig. 4A, B). At the beginning of the night, however, *fba2* displayed an elevated rate of respiration which, within 4 h, decreased to match the wild type. We tested whether *fba2* mutant plants were able to respond to increases in light and CO_2_ concentration in terms of an increased photosynthetic CO_2_ assimilation (see Supplementary Fig. S3). Increasing the light intensity from 150 to 500 μmol m^−2^ s^−1^ led to an 80% increase in CO_2_ assimilation rate in the wild type, but a smaller, 40% increase in *fba2*. Subsequent elevation of the CO_2_ concentration from 380 to 1000 ppm yielded further increases, but again, smaller increases in *fba2*, such that under these conditions, *fba2* photosynthesis was less than 60% that of the wild type. Interestingly, the *fba3* mutant had a decreased photosynthetic rate throughout the day and a decreased respiration rate throughout the night (Fig. 5A). We also tested the capacity of this mutant to respond to increases in light and CO_2_ (Supplementary Fig. S3), but there was almost no change in its photosynthetic carbon assimilation rate in either high light or combined high light and high CO_2_. Under these conditions, *fba3* photosynthesis was less than 10% that of the wild type.

Next, we measured starch and neutral sugars (glucose, fructose, and sucrose) over a diel cycle (Fig. 5B). In *fba1*, there were only minor differences compared with the wild type, with the content of glucose and sucrose being slightly lower during the night. In *fba2*, however, starch accumulation at the end of the day was again reduced to 60% that of the wild type. Instead, the *fba2* mutant accumulated high amounts of glucose and fructose during the day, suggesting elevated production and degradation of sucrose, even though sucrose contents were similar to that of the wild type. The sum of starch and sugars that accumulated in *fba1* during the course of the day was similar to the wild type, but in *fba2* its was 20% lower. During the night, starch degradation in *fba2* was slower than in the wild type, such that the reserves were only depleted at the end of the night. During the first hours of the night, sugar levels declined rapidly in *fba2*, correlating with the period of high respiration. In the second half of the night, both sucrose and glucose in *fba2* dropped below wild-type levels.

The *fba3* mutant was the most remarkable, exhibiting a strong starch-excess phenotype, despite its lower photosynthetic rate. At the end of the day, starch was four times higher than in the wild type, and these levels remained high throughout the night. In addition, the amounts of all the three soluble sugars were much higher than in the wild type and, although levels did decline at night, the diel dynamics in these carbohydrate pools was largely lost. Furthermore, high sugar levels at night in *fba3* were not accompanied by a high respiration rate.

### Profiling reveals marked disturbances in metabolite levels in *fba2* and *fba3* mutants

**Fig. 6.**
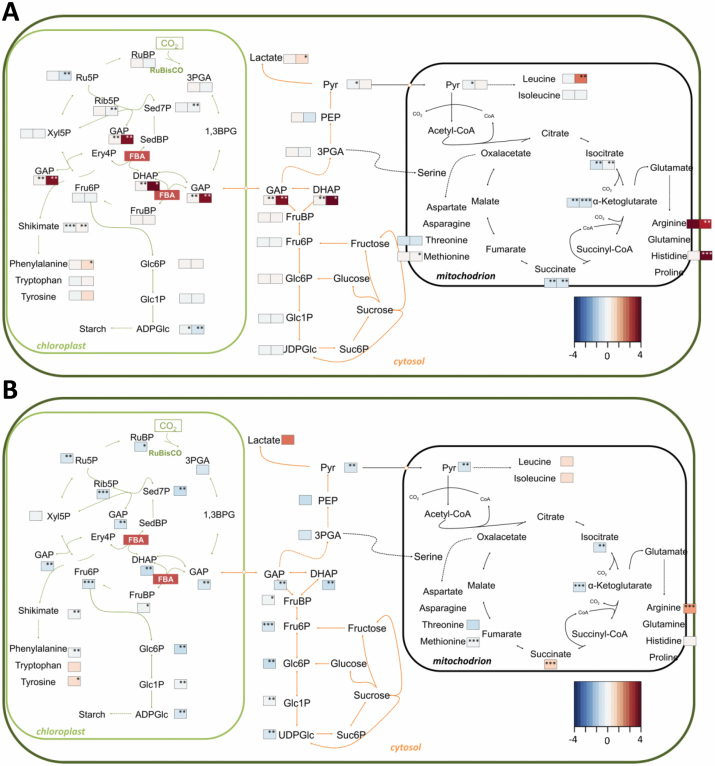
Metabolite levels in the shoots of mutants lacking plastidial *fba* isoforms. Schematic representation of central carbohydrate and amino acid metabolism in photosynthetic cells. Mean metabolite values (*n*=3 biological replicates), normalized to the wild type, are visualized as a fold-change (FC) heatmap. (A) *fba1*-1 (left-hand heatmap boxes) and *fba2*-1 (right-hand heatmap boxes) mutants, and (B) *fba3*-1 mutants. *P≤0.1, **P≤0.05, ***P≤0.01: significant changes in the metabolite level compared with the respective wild type, two-tailed *t*-test. See also Supplementary Table S7. 1,3BPG, 1,3-bisphosphoglycerate; DHAP, dihydroxyacetone phosphate; Ery, erythrose; FBA, fructose-1,6-bisphosphate aldolase; Fru, fructose; GAP, glycerylaldehyde 3-phosphate; Glc, glucose; PEP, phosphoenolpyruvate; 3PGA, 3-phosphoglycerate; Pyr, pyruvate; Rib, ribose; Ru, ribulose; Sed, sedoheptulose; Suc, sucrose; Xyl, xylulose.

We investigated the metabolic changes induced by the knock-out of the individual FBAs using UHPLC-MS/MS (Fig. 6). Metabolites profiled included phosphorylated monosaccharides, Calvin–Benson cycle and glycolytic intermediates, as well as organic acids, amino acids, nucleotides, and energetic intermediates. PCA of the metabolite profiles of shoots harvested midway through the photoperiod robustly separated *fba2* and *fba3* into distinct clusters, whereas both wild-type ecotypes and the *fba1* mutants clustered together (see Supplementary Fig. S4). The metabolite levels in the leaves of *fba1* mutants were similar to the wild type with few exceptions. In the leaves of *fba2*, however, the levels of the triose-phosphates—substrates for FBA in the Calvin–Benson cycle—were elevated compared with the wild type, while FruBP remained unaltered. Ribose 5-phosphate and ribulose 5-phosphate were decreased in *fba2* relative to the wild type, but xylulose 5-phosphate and RuBP were unaltered. ADPglucose was also low in *fba2*, while UDPglucose levels were normal (Fig. 6A; Supplementary Table S7). In *fba3*, the metabolite levels in the leaves were divergent from the other mutants and the wild type. Although the levels of starch and free sugars were very high in this mutant (Fig. 5), the amounts of most intermediates of the Calvin–Benson cycle and central metabolism were lower than in the wild type, as were the amounts of several amino acids and adenylates (Fig. 6; Supplementary Table S7).

**Fig. 7.**
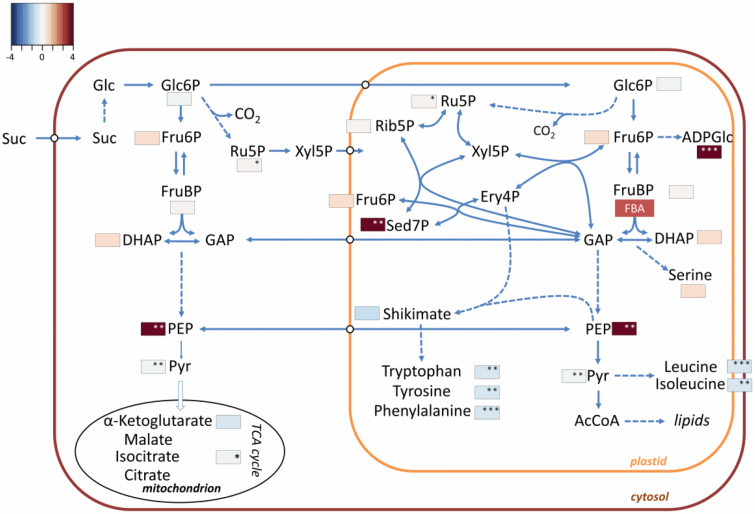
Metabolite levels in the roots of the *fba3* mutant. Schematic representation of central carbohydrate and amino acid metabolism in heterotrophic cells. Mean metabolite values (*n*=4 biological replicates), normalized to the wild type, are visualized as a fold-change (FC) heatmap. *P≤0.1, **P≤0.05, ***P≤0.01: significant changes in the metabolite level in *fba3* roots compared with the wild type, two-tailed *t*-test. See also Supplementary Table S8. AcCoA, acetyl-CoA; DHAP, dihydroxyacetone phosphate; Ery, erythrose; FBA, fructose-1,6-bisphosphate aldolase; Fru, fructose; GAP, glycerylaldehyde 3-phosphate; Glc, glucose; PEP, phosphoenolpyruvate; 3PGA, 3-phosphoglycerate; Pyr, pyruvate; Rib, ribose; Ru, ribulose; Sed, sedoheptulose; Suc, sucrose; Xyl, xylulose.

Since *FBA3* is expressed in the roots, we also profiled the root metabolites of the *fba3* mutant. In non-photosynthetic tissues, carbon is supplied as phloem-transported sugar rather than via photosynthesis, and plastidial FBA is proposed to act in a glycolytic capacity. Interestingly, the levels of FruBP and of DHAP were normal in *fba3* (Fig. 7; Supplementary Table S8). However, there were significant changes in closely related metabolites: sedoheptulose-7-phosphate (Sed7P) and PEP were elevated, while amino acids derived from the shikimate pathway (tyrosine, tryptophan, and phenylalanine) were decreased in *fba3* relative to the wild type. ADPglucose—undetectable in the wild type—was also increased in the *fba3* roots.

### Evidence for a primary defect in vascular function in the *fba3* mutant shoots

Because *fba3* mutants had extremely high carbohydrate concentrations in the leaves and *FBA3* is expressed in the vasculature, we reasoned that the mutants may have a deficiency in vascular structure and/or function that limits photoassimilate export. Examination of the vascular pattern of *fba3* leaves did not reveal any differences from the wild type, although the leaves were much smaller (see Supplementary Fig. S5). Since the amino acid products of the shikimate pathway, which serve as precursors for lignin biosynthesis, were lower in *fba3* than in wild-type roots, we investigated whether any defects in lignification could be observed. Staining cleared roots and shoots with Basic Fuchsin and examining stem sections for lignin autofluorescence did not reveal any apparent decreases in lignification in *fba3* relative to the wild type (Supplementary Fig. S5).

**Fig. 8.**
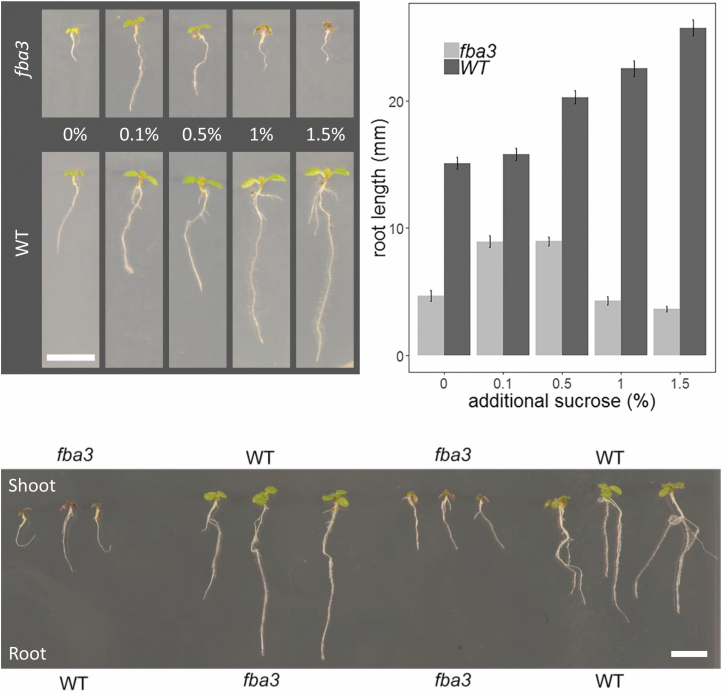
Root growth phenotype of the *fba3* mutant and its rescue by micrografting. (A) Representative seedling phenotypes of the *fba3*-1 mutant and its L*er* wild type grown on agar plates without or with exogenous sucrose (0.1, 0.5, 1, and 1.5% (w/v)). (B) Mean root length (±SE; *n*=12 biological replicates) for plants grown as in (A). (C) Phenotype of chimeric plants where *fba3* shoots are grafted onto wild-type roots and vice versa. Self-grafted *fba3* and wild-type plants serve as controls. The genotype of the roots was determined by PCR on extracted genomic DNA to re-confirm that they were not lateral roots produced at or above the graft point. Scale bar: 1 cm.

Next, we analysed root growth. Homozygous *fba3* plants grown on agar plates had much shorter roots than the wild type (Fig. 8A, B). Provision of exogenous sucrose, while increasing root growth of the mutant and the wild type at low concentrations, failed to rescue the mutant phenotype completely. In fact, higher sugar concentrations exacerbated the short root phenotype of *fba3* despite further stimulating wild-type root growth (Fig. 8A, B). To explore the *fba3* root-growth phenotype further, micro-grafting was carried out to determine if the dysfunction affected primarily the export of substances from the shoot or their utilization by the root. Chimeric plants where *fba3* shoots were grafted onto wild-type roots resembled the *fba3* mutant and control plants where mutant shoots were re-grafted onto their roots (Fig. 8C). In contrast, when wild-type shoots were grafted onto *fba3* roots, the short root phenotype of *fba3* was rescued. Collectively, these data show that although the loss of *fba3* affects root metabolite levels, root growth is either restricted by a primary defect in the shoot or that export from the wild-type shoot can compensate—presumably via long distance transport of more than just sugars—for the metabolic deficiency in the root.

## Discussion

The plastidial localizations of FBA1, FBA2, and FBA3 were previously confirmed experimentally in Arabidopsis (Vidi *et al.*, 2006; Ytterberg *et al.*, 2006; Lu *et al.*, 2012). Our analysis of their gene expression patterns and our functional studies show that FBA1 and FBA2 together fulfil the canonical, essential role of FBA within the Calvin–Benson cycle in the leaf mesophyll. In contrast, FBA3 has a distinct, important role in the leaf vasculature and root. Phylogenetic analyses suggest that the genomes of all plant species analysed encode multiple isoforms, but also suggest that an ancient gene duplication led to an angiosperm-specific family containing AtFBA1 and AtFBA2 proteins. Thus, in more primitive plants such as the Selaginellaceae and Bryophyta, it is presumably homologs of FBA3 that function in the Calvin–Benson cycle.

### Chloroplastic FBA activity in photosynthetic tissues is provided by FBA2 and FBA1

The Arabidopsis *FBA1* and *FBA2* genes are the result of a relatively recent segmental genome duplication event and represent partially redundant isoforms. Both have similar expression patterns and appear to function in photosynthesis. *FBA2* is the more highly expressed of the two genes in leaves (see Supplementary Fig. S1; Fig. 3A) and contributes the majority of the leaf FBA activity, judging from the ca. 70% reduction in the *fba2* mutants (Fig. 3E). A complete loss of activity would not be expected for any of the single mutants, but previous estimates suggest that chloroplastic FBA contributes 85–95% of the total activity in leaves (Schnarrenberger and Krüger, 1986; Haake *et al.*, 1998). Presumably, FBA1 and FBA3 also contribute to the total leaf activity, but as little or no difference in total activity could be measured in their mutants, their individual contribution is probably small, because of their lower expression level, smaller expression domain, or lower specific activity.

The strong phenotype of the *fba2* mutant—slightly pale in appearance, slow growth, and a shift in partitioning of assimilates away from leaf starch towards excess sugar production—is consistent with previous studies where plastidial FBA expression was repressed in potato by antisense RNA expression (Haake *et al.*, 1998). Strong repression similarly resulted in small pale plants with low levels of leaf starch. It is notable that, despite its pale appearance, *fba2* chlorophyll content was similar to the wild type. Furthermore, daytime CO_2_ assimilation rates in *fba2* were unaffected (Fig. 5A), unless high light and CO_2_ conditions were imposed (see Supplementary Fig. S3), where the low FBA activity presumably limits the regenerative capacity of the Calvin–Benson cycle. These observations suggest that the low growth rate observed for this mutant under normal growth-room conditions may be caused primarily by the altered photoassimilate partitioning (Fig. 5B). It is well established that leaf starch acts as an important buffer against the diel changes in photosynthesis (Stitt and Zeeman, 2012). Mutants affecting either starch biosynthesis or starch remobilization have strongly decreased growth rates (unless grown in very long photoperiods) and exhibit reduced allocation of assimilates into structural biomass (Kölling et al., 2015b). The precise reason for the shift in partitioning in *fba2* is unclear, but may be due to increased export of triose phosphates from the chloroplast. Triose phosphates were increased in abundance, likely due to the limiting FBA activity. However, the level of 3PGA, the immediate product of Rubisco and a known allosteric activator of the starch biosynthesis pathways, was unchanged. Levels of ADPglucose—the immediate precursor of the starch synthases—were decreased in *fba2*. This suggests that the flux catalysed by the remaining FBA1 was used primarily for the continued operation of the Calvin–Benson cycle, at the expense of starch biosynthesis. In contrast, the increased levels of both free glucose and fructose are indicative of increased sucrose production in the cytosol and turnover in the vacuole.

The loss of the minor isoform, FBA1, had little phenotypic effect, in terms of growth, photosynthesis, or the metabolite profile, suggesting that the activity of the major isoform, FBA2, is sufficient. However, our analyses of segregants derived from a cross between the two single mutants illustrates that FBA1 is critical for viability in the absence of FBA2. Firstly, the *fba1fba2* double mutant was very difficult to obtain due to a high frequency of seed abortion, and the sole double mutant seedling identified was not viable beyond the seedling stage. Secondly, plants with only one copy of either *FBA1* or *FBA2* in the null mutant background of the other gene (i.e. *fba1*^(−/+)^*fba2*^(−/−)^ and *fba1*^(−/−)^*fba2*^(+/−)^, respectively) showed that a single copy of *FBA2* was sufficient to maintain a near wild-type growth rate, whereas losing one copy of *FBA1* exacerbated the already severe *fba2* phenotype (Fig. 4).

All together, these data suggest that FBA1 and FBA2 do not have discrete roles. Rather, both genes contribute to producing more than sufficient activity required for maintaining normal rates of carbon assimilation and a flux towards transitory starch. While a single *FBA2* allele appears sufficient in the standard growth conditions we used, it is likely that under saturating light conditions, photosynthesis may become limited by the low FBA activity, as in the *fba2* mutant (see Supplementary Fig. S3).

### A distinct role of FBA3 in the plastids of non-photosynthetic tissues

Among the three mutants, *fba3* had the most severe phenotype, exhibiting very slow growth (both of the shoots and roots) and the accumulation of excessive carbohydrates in the shoots. Yet *fba3* plants exhibit large reductions in photosynthetic rate and reductions in the levels of many intermediates of photosynthesis and central metabolism, compared with wild-type plants. This severe leaf phenotype is apparent despite (i) *FBA3* expression levels being low (when considered at a whole leaf level) and restricted to phloem-associated cells within the vasculature, and (ii) no measurable change in total FBA activity, either in the shoot or in the root, where *FBA3* expression is higher. The expression pattern of *FBA3* suggests that the accumulation of leaf carbohydrates may result from a deficiency in photoassimilate export, with the reduction of photosynthesis a secondary consequence of excess sugar accumulation, rather than a primary consequence of FBA3 deficiency.

We speculate that *fba3* has a role in the plastids of the phloem-associated cells required for sugar transport to sink tissues. Mutants of sucrose transporters specific to the bundle sheath cells (*sweet11sweet12*) or phloem cells (*suc2*) showed a similar carbohydrate-accumulating phenotypes (i.e. high starch and sugar content in the leaves) (Gottwald *et al.*, 2000; Chen *et al*, 2012). These mutations limit sugar loading and transport to the sink tissues and, like *fba3*, display stunted root growth. However, unlike *fba3*, this root-growth phenotype could be rescued with exogenous supply of sugars. Thus, *fba3* is afflicted by more than just a lack of sugar export to the roots.

In non-photosynthetic tissues, where *FBA3* is expressed, plastidial FBA functions as a glycolytic enzyme. Both glycolysis and the oxidative pentose phosphate pathway in the plastid will be supplied with hexose-phosphates imported via the plastid envelope glucose-phosphate translocator (Kammerer *et al.*, 1998; Niewiadomski *et al.*, 2005). These respiratory pathways serve to support the organelle’s needs for energy and reducing power and drive biosynthetic processes located partially or exclusively in the plastid (including the biosynthesis of fatty acids, numerous amino acids, nucleotides, isoprenoids (via the MEP pathway), amongst others). Substrates for these biosynthesis pathways are withdrawn from different parts of the plastidial respiratory pathways. Flux analyses of heterotrophic cell cultures supplied with glucose have estimated that 50% or more of the metabolized carbohydrate flows through the top part of plastidial glycolysis, including the FBA reaction, to produce triose-phosphates (Masakapalli *et al.*, 2014). Our data show that this conversion is extremely important for normal rates of plant growth. Nevertheless, *fba3* plants are viable, suggesting that metabolite exchange between the cytosol and plastid, via the numerous metabolite transporters located in the plastid envelope, is sufficient to maintain plastidial metabolism above a critical level. Alternatively, it is possible that low levels of *FBA1* and/or *FBA2* expression in the roots allow a limited flux to proceed through the plastidial FBA step.

One enzyme that metabolizes the triose-phosphate products of pFBA in the plastid is glyceraldehyde-3-phosphate dehydrogenase (GAPDH; catalysing the conversion of GAP to 1,3-bisphosphoglycerate while reducing NAD^+^ to NADH), which continues the flux down the glycolytic sequence. Double mutants lacking two plastidial GAPDH isoforms (GAPCp1 and GAPCp2) also have reduced shoot and root growth and accumulate more soluble sugars in the leaves, (Muñoz-Bertomeu *et al.*, 2009). The genes encoding these isoforms have a similar expression patterns to *FBA3* and are important for allowing serine biosynthesis via the so-called phosphorylated pathway, which uses 3PGA as a substrate (Ho and Saito, 2001). However, while serine was reportedly decreased in *gapcp1 gapcp2* double mutants (Muñoz-Bertomeu *et al.*, 2009), our metabolite analyses did not reveal a change in serine levels in *fba3* compared with the wild type, so it seems unlikely to be the cause of the overall defects in plant growth. That said, the levels other amino acids were affected. Both leucine and isoleucine were significantly decreased in *fba3* roots, and it was shown that isoleucine deficiency can impair root development (Yu *et al.*, 2013). The aromatic amino acids tyrosine, tryptophan, and phenylalanine were also significantly lower in *fba3* than in the wild type (the mean value for shikimate—an intermediate in aromatic amino acid production—was considerably lower in *fba3* roots than in the wild type, but this result was not statistically significant due to high variation in the latter).

A limitation in triose phosphates and metabolites in the lower half of plastid glycolysis, caused by FBA3 deficiency, could explain these reduced amino acid levels. For the production of leucine and isoleucine, pyruvate is required, which was lower in *fba3* than in the wild type. For the shikimate pathway, Ery4P is needed. This in turn is produced by the actions of transaldolase or transketolase, both of which require GAP as one of their substrates. Unfortunately, neither GAP nor Ery4P itself was measurable in our root extracts. However, in the case of transaldolase, the other substrate, Sed7P, was markedly increased in *fba3* roots compared with the wild type. This is consistent with a limitation in GAP availability. Furthermore, the increase in PEP is also consistent with this hypothesis since PEP is reacted with Ery4P to create 3-deoxy-D-arabino-heptulosonate-7-phosphate (DAHP)—the first step in the shikimate pathway. Although the shikimate pathway also provides substrates for lignin production (Henkes *et al.*, 2001; Maeda and Dudareva, 2012), we could not detect any reductions in the degree of lignification in *fba3* plants compared with the wild type (see Supplementary Fig. S5). This is perhaps not surprising for the leaf, since most detected lignin was in the xylem, where FBA1 and FBA2 are expressed. Thus, a deficiency in phloem-localized FBA3 would be unlikely to affect xylem lignification. Alternatively, with the very slow rate of growth, normal lignin levels may eventually be produced even if there is a limitation of this pathway.

While our metabolite measurements in the root gave insight into the role of plastidial FBA in this heterotrophic tissue, our grafting experiments yielded an important result, showing that *fba3* plays a critical role in the areal parts of the plant. The fact that the wild-type shoot could rescue the slow growth of the *fba3* roots suggests that the metabolic deficiency in the root can be overcome by metabolites supplied via the phloem from the shoot—presumably both sugars from the mesophyll and amino acids synthesized in the *FBA3*-expressing shoot vascular tissues. In contrast, wild type roots failed to grow normally when grafted onto *fba3* shoots. We propose that this is due to the failure of the transport tissues of the shoot to provide sugars to the root. Collectively, these results point at a primary deficiency in the leaf vasculature of the *fba3* mutant. We suggest that, in the leaf vasculature, as in the root, FBA3 deficiency limits the production of essential compounds in the plastid, including amino acids. The symplasmic isolation of the companion cells and the phloem may mean that these compounds cannot be provided by chloroplastic metabolism in the rest of the leaf. We further suggest that the metabolic limitation results in phloem dysfunction, which in turn prevents sugar export, causing a buildup of carbohydrates in the leaf and eventually end-product repression of photosynthesis.

In conclusion, we have shown that FBA1 and FBA2 function in the Calvin–Benson cycle and that this step is, as expected, essential. Meanwhile, FBA3 has a distinct, yet key role in plastid respiration in non-photosynthetic tissues of the root and the shoot. Our analyses provide important clues about the specific processes compromised by the loss of FBA3, although further experiments will be needed to test our conclusions. Such experiments could include the cell-type specific complementation of the *fba3* mutant using the wild type gene driven by cell-type specific promoters (e.g. for the companion cells or for the root). Alternatively, a strategy similar to that taken by Flores-Tornero *et al.* (2017) could be used, whereby the triose phosphate translocator could be expressed under the *FBA3* promoter to assess whether the plastidial FBA reaction could be bypassed, as was possible for the *gapcp1 gapcp2* double mutant.

## Supplementary data

The following supplementary data are available at *JXB* online.

Fig. S1. Expression and protein levels of FBA1, FBA2, and FBA3 throughout the plant.

Fig. S2. Opened siliques of *fba1*^(−/−)^*fba2*^(−/+)^ and *fba1*^(−/+)^*fba2*^(−/−)^ mutant plants revealing aborted seeds.

Fig. S3. Changes in photosynthetic rate in *fba2*-1, *fba3*-1, and their respective wild types in response to elevated light and CO_2_.

Fig. S4. PCA of the shoot metabolomic measurements of the plastidial *fba* mutants and their corresponding wild types.

Fig. S5. Comparison of vascular patterns and xylem lignification in the *fba3* mutant and its corresponding wild type.

Table S1. Primers sequences used in this study.

Table S2. Gradients for high-performance anion exchange chromatography used for sugar measurements.

Table S3. Gradients for ion-pair reversed-phase chromatography used during the UHPLC-MS/MS metabolite measurements.

Table S4. Amino acid similarity between the plastidial FBA proteins.

Table S5. Growth of the plastidial *fba* mutant lines and respective wild-type lines 30 d after germination.

Table S6. Segregation of genotypes in the F2 generation of the cross between *fba1* and *fba2* mutants.

Table S7. Metabolite contents in the aerial parts of the plastidial *fba* mutants and their respective wild types.

Table S8. Metabolite contents in the roots of *fba3* and its wild type.

erab099_Supplementary_Data

## Data Availability

All raw data underlying the results presented here are available on request. Sequence data of the Arabidopsis genes studied in this article can be found in TAIR (www.arabidopsis.org) under the following accession numbers: FBA1 (AT2G21330), FBA2 (AT4G38970), FBA3 (AT2G01140).
