# Supplementary material for: Distinct plastid fructose bisphosphate aldolases function in photosynthetic and non-photosynthetic metabolism in Arabidopsis
Source: J Exp Bot. 2021 Mar 4;72(10):3739–55. doi: 10.1093/jxb/erab099 (PMC8628874; doi:10.1093/jxb/erab099)
Supplement: erab099_Supplementary_Data [file erab099_Supplementary_Data.pdf]

Supplementary information – Carrera et al., Plastid Aldolase functions

Supplementary Table S1. Primer sequences used in this study. Primers for genotyping *fba* mutants, quantifying expression of the respective *FBA* genes by RT-qPCR, and amplification and Gateway® recombinant cloning of *FBA* promoters. Primers for genotyping were designed upstream (FW) and downstream (RV) of the insertion and used together to detect the wild-type allele. FW primers were also used together with the appropriate T-DNA specific primer to detect the mutant alleles.

Name	Genome location	Sequence (5' → 3')
fba1-1 FW	*AtFBA1* (At2g21330)	TTGTTGGGAATTGTCGATTTC
fba1-1 RV	*AtFBA1* (At2g21330)	CTTGTTGGTAGTAAGCAGCGG
SALK LBB4	TDNA LB	GCGTGGACCGCTTGCTGCAACT
fba1-2 FW	*AtFBA1* (At2g21330)	GCTCGCTACGCAGCTATTTC
fba1-2 RV	*AtFBA1* (At2g21330)	TTTTGAGCTCAGAGAGCATAATGT
SAIL LB1	TDNA LB	GCCTTTTCAGAAATGGATAAATAGCCTTGCTTCC
fba2-1 FW	*AtFBA2* (At4g38970)	TCCATCCAACAAGATCTCTGG
fba2-1 RV	*AtFBA2* (At4g38970)	TGTTCTGTTTTGCCCTGTTTC
fba2-2 FW	*AtFBA2* (At4g38970)	GTGCCTTTTGAATGGGAATG
fba2-2 RV	*AtFBA2* (At4g38970)	CAAGTTGAGTGTTGCCTCC
fba3-1 FW	*AtFBA3* (At2g01140)	GTGCTTACTCCGACGAGCTT
fba3-1 RV	*AtFBA3* (At2g01140)	CGTTGTTCTGTGCCAAGTAG
GT12795.Ds5	TDNA LB	TACCTCGGGTTCGAAATCGAT
fba3-2 FW	*AtFBA3* (At2g01140)	TCGTTTTGCCAAGTGGTATG
fba3-2 RV	*AtFBA3* (At2g01140)	GAGAGAACGAGGATGCCAAG
FBA1_FW_qPCR	*AtFBA1* (At2g21330)	GAAGGTATTCTCCTGAAGCCA
FBA1_RV_qPCR	*AtFBA1* (At2g21330)	CACCAGACAAGAACATGATTCC
FBA2_FW_qPCR	*AtFBA2* (At4g38970)	TGTCATGTTTGAAGGTATCCTC
FBA2_RV_qPCR	*AtFBA2* (At4g38970)	TTCAAGTTGAGTGTTGCCTC
FBA3_FW_qPCR	*AtFBA3* (At2g01140)	CAGAACATGGCAAGGCAAGC
FBA3_RV_qPCR	*AtFBA3* (At2g01140)	TCCCTCGGCTGAGTATTTGC
FBA3_FW2_qPCR	*AtFBA3* (At2g01140)	CCGGTGCTTACTCCGACGAG
FBA3_RV2_qPCR	*AtFBA3* (At2g01140)	TAATCGCCGAGGCCAGGAGT
HKG_YSL_FW_qPCR	*AtYLS8* (At5g08290)	ATGACTGGGATGAGACCTGTATGC
HKG_YSL_RV_qPCR	*AtYLS8* (At5g08290)	CATGGTGTTGAAGTCTGGAACCTC
HKG_ACT_FW_qPCR	*ACTIN2* (At3g18780)	TCTTCCGCTCTTTCTTTCCAAGC
HKG_ACT_RV_qPCR	*ACTIN2* (At3g18780)	ACCATTGTCACACACGATTGGTTG
HKG_GDH_FW_qPCR	*GAPC2* (AT1G13440)	AGGTGGAAGAGCTGCTTCCTTC
HKG_GDH_RV_qPCR	*GAPC2* (AT1G13440)	GCAACACTTTCCCAACAGCCT
proFBA1_FW01	pro*AtFBA1*	GGGGACAACTTTGTATAGAAAAGTTGAGCGTTGTTTTCATGTTGGAG
proFBA1_RV01	pro*AtFBA1*	GGGGACTGCTTTTTTGTACAAACTTGTCGTGCTTTTCGGTGGTTTG
proFBA2_FW01	pro*AtFBA2*	GGGGACAACTTTGTATAGAAAAGTTGAGACGCAGCAGAGGTTTCTC
proFBA2_RV01	pro*AtFBA2*	GGGGACTGCTTTTTTGTACAAACTTGCCTTATCTCTCACTCCTCCCTC
proFBA3_FW01	pro*AtFBA3*	GGGGACAACTTTGTATAGAAAAGTTGAAGATCTACCGAAAGCAAGG
proFBA3_RV01	pro*AtFBA3*	GGGGACTGCTTTTTTGTACAAACTTGATCACCTACTCCCACCACC

Supplementary Table S2. Gradients for high-performance anion exchange chromatography used for sugar measurements. Eluent A, 100 mM NaOH; Eluent B, 150 mM NaOH and 500 mM CH_3COONa.

Time [min]	Flow rate [ul/min]	%A	%B
0	500	100	0
15	500	100	0
26.5	500	20	80
32.5	500	20	80
33	500	100	0
40	500	100	0

Supplementary Table S3. Gradients for ion-pair reversed-phase chromatography used during the UHPLC-MS/MS metabolite measurements after Büscher et al. (2010). Eluent A: 10 mM tributylamine, 15 mM acetic acid, 5 µM phosphoric acid, 5% (v/v) methanol in water; Eluent B: 100% isopropanol.

Time [min]	Flow rate [ul/min]	%A	%B
0	400	100	0
5	400	100	0
10	400	98	2
11	350	91	9
16	250	91	9
18	250	75	25
19	150	50	50
25	150	50	50
26	150	100	0
32	400	100	0
40	400	100	0

Supplementary Table S4. Amino acid similarity between the plastidial FBA proteins.

	FBA1	*FBA2*	*FBA3*
FBA1	100		
FBA2	94.9	100	
FBA3	84.8	85	100

Supplementary Table S5. Growth of the plastidial *fba* mutant lines and respective wild-type lines 30 days after germination. Leaf area, fresh weight, dry weight and chlorophyll content were determined. Values are the means ± SD of at least 6 biological replicates, with the exception of the chlorophyll which is the mean of 5 biological replicates ± SD. Statistically significant differences from the respective wild types are indicated with one, two and three asterisks (p ≤ 0.1, 0.05, and 0.01, respectively, 2-tailed t-test).

Genotype	Leaf area (cm^2)	Fresh weight (mg)	Dry weight (mg)	FW/DW	Chlorophyll (mg m^{-2})
fba1-1	6.96 ±1.19	168 ± 21	15 ± 2	11.50	190 ± 26*
fba1-2	7.49 ±1.91	205 ± 51	19 ± 4	11.09	158 ± 16
fba2-1	1.42 ± 0.19***	28 ± 5***	3 ± 1***	9.70	176 ± 7*
fba2-2	1.21 ± 0.23***	23 ± 5***	2 ± 1***	11.28	142 ± 4**
fba3-1	0.48 ± 0.06***	12 ± 2***	2 ± 1***	7.55	179 ± 47
fba3-2	1.4 ± 0.372***	22 ± 3***	3 ± 1***	8.09	127 ± 51
WT *(*Col-0)	7.35 ± 1.5	171 ± 46	15 ± 4	11.42	164 ± 11
WT (L*er*)	7.42 ± 1.51	187 ± 50	17 ± 5	11.03	189 ± 8

Supplementary Table S6. Segregation of genotypes in the F2 generation of the cross between *fba1* and *fba2* mutants. Observed percentages are given, with expected percentages in parentheses (n = 268). Values in red are higher than expected and those in blue are lower than expected (p = 0.02; X^2 test).

	FBA2FBA2	***fba2FBA2***	***fba2fba2***
FBA1FBA1	**11.2** (6.25)	11.9 (12.5)	5.2 (6.25)
fba1FBA1	**18.7** (12.5)	**34** (25)	**3** (12.5)
fba1fba1	7.1 (6.25)	**8.6** (12.5)	**0.4** (6.25)

Supplementary Table S7. Metabolite contents (nmol/g FW) in the aerial parts of *fba1*, *fba2*, *fba3* and the respective wild types. Values are the means ± SD of three biological replicates. Values highlighted in red indicate significant increases and those in blue indicate significant decreases. Light, medium and dark colours represents p ≤ 0.1, 0.05, and 0.01, respectively.

Metabolite	*fba1*	*fba2*	WT (Col)	*fba3*	WT (Ler)
Adenine	5 ± 3	5 ± 0	5 ± 1	23 ± 14	3 ± 1
ADP	154 ± 16	138 ± 37	188 ± 20	16 ± 6	123 ± 39
ADP-Glucose	9 ± 0	5 ± 1	11 ± 2	4 ± 0	8 ± 2
AMP	116 ± 9	78 ± 27	102 ± 2	19 ± 16	60 ± 8
ATP	359 ± 44	512 ± 69	819 ± 119	87 ± 61	538 ± 143
Arginine	138 ± 70	97 ± 24	24 ± 11	136 ± 3	52 ± 9
DHAP	265 ± 16	1276 ± 434	210 ± 17	20 ± 16	157 ± 43
FAD	15 ± 0	16 ± 2	16 ± 2	9 ± 2	17 ± 1
Fructose 1,6-BP	179 ± 24	192 ± 32	164 ± 21	74 ± 5	88 ± 9
Fructose 6-P	477 ± 13	505 ± 44	509 ± 36	138 ± 49	441 ± 67
Glucose 1-P	167 ± 14	185 ± 17	186 ± 13	62 ± 7	79 ± 4
Glucose 6-P	624 ± 103	702 ± 118	586 ± 50	29 ± 22	308 ± 97
Glyceraldehyde 3-P	12 ± 1	46 ± 9	10 ± 1	2 ± 0	6 ± 1
Glycerate	635 ± 53	440 ± 137	700 ± 59	590 ± 56	855 ± 44
Histidine	73 ± 11	334 ± 44	56 ± 14	52 ± 17	44 ± 4
Isocitrate	421 ± 19	606 ± 31	775 ± 39	260 ± 26	652 ± 85
Isoleucine	100 ± 17	120 ± 31	121 ± 30	149 ± 41	99 ± 13
α-Ketoglutarate	342 ± 13	238 ± 12	676 ± 75	176 ± 2	556 ± 26
Lactate	645 ± 45	870 ± 173	584 ± 119	1772 ± 1116	579 ± 126
Leucine	90 ± 20	297 ± 24	102 ± 28	114 ± 26	80 ± 4
Methionine	147 ± 15	190 ± 29	138 ± 7	141 ± 8	196 ± 11
Phenylalanine	290 ± 47	456 ± 81	267 ± 90	152 ± 29	207 ± 23
Phosphoenolpyruvate	8 ± 4	3 ± 2	6 ± 2	0.1 ± 0.2	2 ± 1
3-Phosphoglycerate	360 ± 40	316 ± 186	355 ± 14	77 ± 2	195 ± 66
Pyruvate	604 ± 41	811 ± 83	785 ± 88	426 ± 132	760 ± 29
Ribose 5-P	38 ± 6	24 ± 3	37 ± 2	8 ± 3	37 ± 4
Ribulose 5-P	195 ± 5	73 ± 9	206 ± 27	99 ± 46	237 ± 37
Ribulose 1,5-BP	140 ± 37	82 ± 57	115 ± 13	32 ± 8	68 ± 21
Sedoheptulose 7-P	338 ± 16	259 ± 44	388 ± 58	50 ± 32	296 ± 86
Shikimate	137 ± 4	215 ± 21	156 ± 1	109 ± 2	135 ± 7
Succinate	352 ± 10	509 ± 60	1356 ± 297	1582 ± 144	769 ± 31
Threonine	750 ± 443	632 ± 138	1397 ± 657	258 ± 81	1532 ± 1368
Tryptophan	32 ± 6	44 ± 7	35 ± 12	49 ± 10	34 ± 2
Tyrosine	27 ± 5	44 ± 16	28 ± 7	54 ± 13	31 ± 2
UDP-Glucose	407 ± 40	338 ± 29	458 ± 86	189 ± 7	321 ± 9
Uridine	16 ± 2	24 ± 9	20 ± 4	85 ± 32	24 ± 6
Xylulose 5-P	26 ± 4	24 ± 8	27 ± 2	14 ± 4	23 ± 2

Supplementary Table S8. Metabolite contents (nmol/g FW) in the roots of *fba3* and its wild type. Values are the means ± SD of three (wild type) or four (*fba3*) biological replicates. Values in red indicate significant increases and those in blue indicate significant decreases. Light, medium and dark colours represents p ≤ 0.1, 0.05, and 0.01, respectively. n.d., not detected.

Metabolite	*fba3*			WT (Ler)		
Aconitate	4	±	1	8	±	1
Adenine	63	±	33	210	±	100
ADP	57	±	20	41	±	9
ADP-Glucose	3	±	2	n.d.		
AMP	59	±	25	83	±	11
Arginine	92	±	28	62	±	11
DHAP	12	±	5	8	±	1
FAD	5	±	2	8	±	1
Fructose 1,6-BP	350	±	87	253	±	31
Fructose 6-P	223	±	91	126	±	8
Glucose 1-P	19	±	6	34	±	3
Glucose 6-P	256	±	121	279	±	26
Glycerate	367	±	221	2306	±	2142
Glycerol 3-P	37	±	17	51	±	5
Histidine	89	±	24	144	±	42
Isocitrate	26	±	7	40	±	8
Isoleucine	94	±	44	214	±	34
α-Ketoglutarate	79	±	31	164	±	64
Lactate	682	±	377	581	±	188
Leucine	115	±	48	275	±	31
Methionine	17	±	7	29	±	6
Phenylalanine	20	±	6	45	±	7
Phosphoenolpyruvate	2	±	1	n.d.		
Proline	230	±	36	158	±	23
Pyruvate	68	±	7	98	±	14
Ribose 5-P	11	±	4	11	±	3
Ribulose 5-P	3	±	1	2	±	1
Sedoheptulose 7-P	531	±	269	12	±	3
Serine	1735	±	629	1026	±	276
Shikimate	9	±	2	52	±	27
Threonine	475	±	100	278	±	22
Tryptophan	14	±	6	27	±	4
Tyrosine	28	±	11	63	±	15
UDP-Glucose	216	±	76	318	±	38
Uracil	3	±	2	5	±	1
Uridine	15	±	9	60	±	32

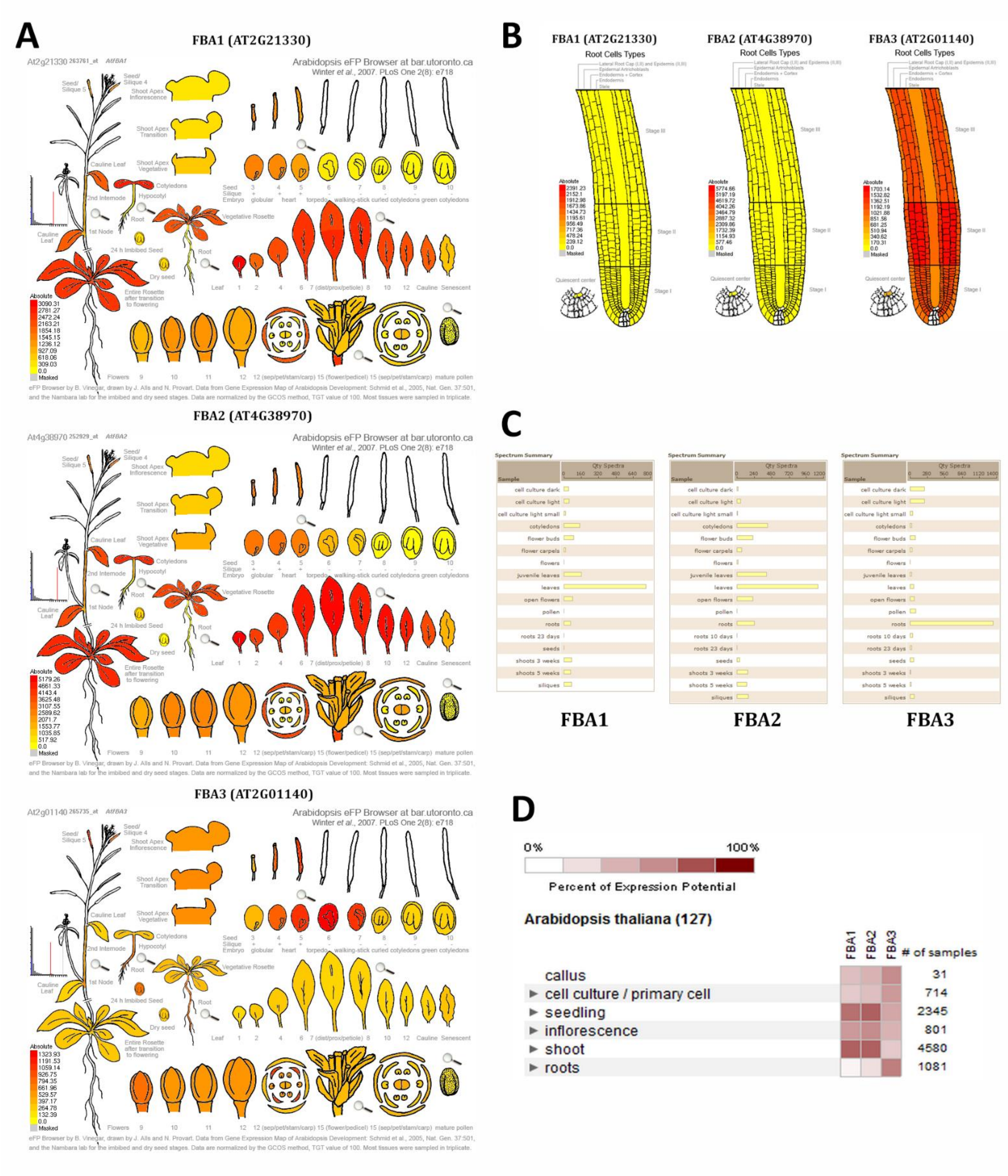
A
FBA1 (AT2G21330)
FBA2 (AT4G38970)
FBA3 (AT2G01140)
Arabidopsis eFP Browser at bar.utoronto.ca
Winter et al., 2007. PLoS One 2(8): e718
B
FBA1 (AT2G21330)
FBA2 (AT4G38970)
FBA3 (AT2G01140)
Root Cells Types
Stage III
Stage II
Stage I
Quiescent center
C
Spectrum Summary
Qty Spectra
Sample
cell culture dark
cell culture light
cell culture light small
cotyledons
flower buds
flower carpels
flowers
juvenile leaves
leaves
open flowers
pollen
roots
seeds
shoots 3 weeks
shoots 5 weeks
siliques
FBA1
FBA2
FBA3
D
0%
100%
Percent of Expression Potential
Arabidopsis thaliana (127)
FBA1
FBA2
FBA3
# of samples
callus 31
cell culture / primary cell 714
seedling 2345
inflorescence 801
shoot 4580
roots 1081

Supplementary information – Carrera et al., Plastid Aldolase functions

Supplementary Figure S1. Expression and protein levels of *FBA1*, *FBA2* and *FBA3* throughout the plant. A) Expression levels of *FBA1* (top panel), *FBA2* (middle panel) and *FBA3* (lower panel) in different plant tissues, particularly of the shoot and reproductive organs B) Expression level of *FBA1*, *FBA2* and *FBA3* in the root tip. Data displays in A and B were obteined via the *Arabidopsis* eFP browser (Winter et al., 2007). C) Number of identified peptides detected in proteomic experiment (pep2pro; Baerenfaller et al., 2011). D) Expression level in different tissues (Genevestigator; Zimmermann et al., 2004)

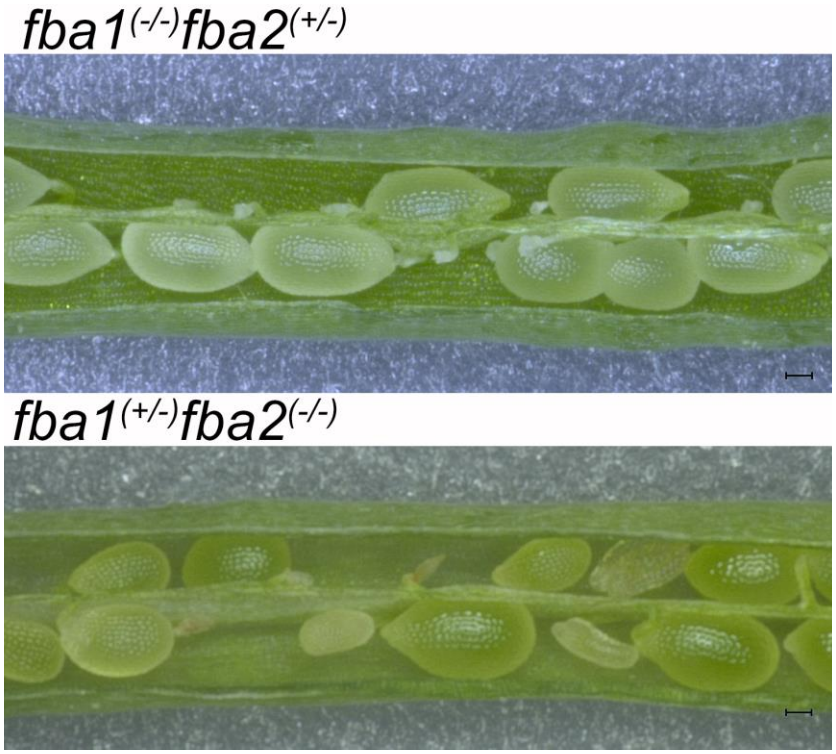

Supplementary Figure S2. Opened siliques of *fba1*$^{(-/-)}$*fba2*$^{(-/+)}$ (top) and *fba1*$^{(-/+)}$*fba2*$^{(-/-)}$ (bottom) mutant plants revealing aborted seeds. Scale bar = 100 µm.

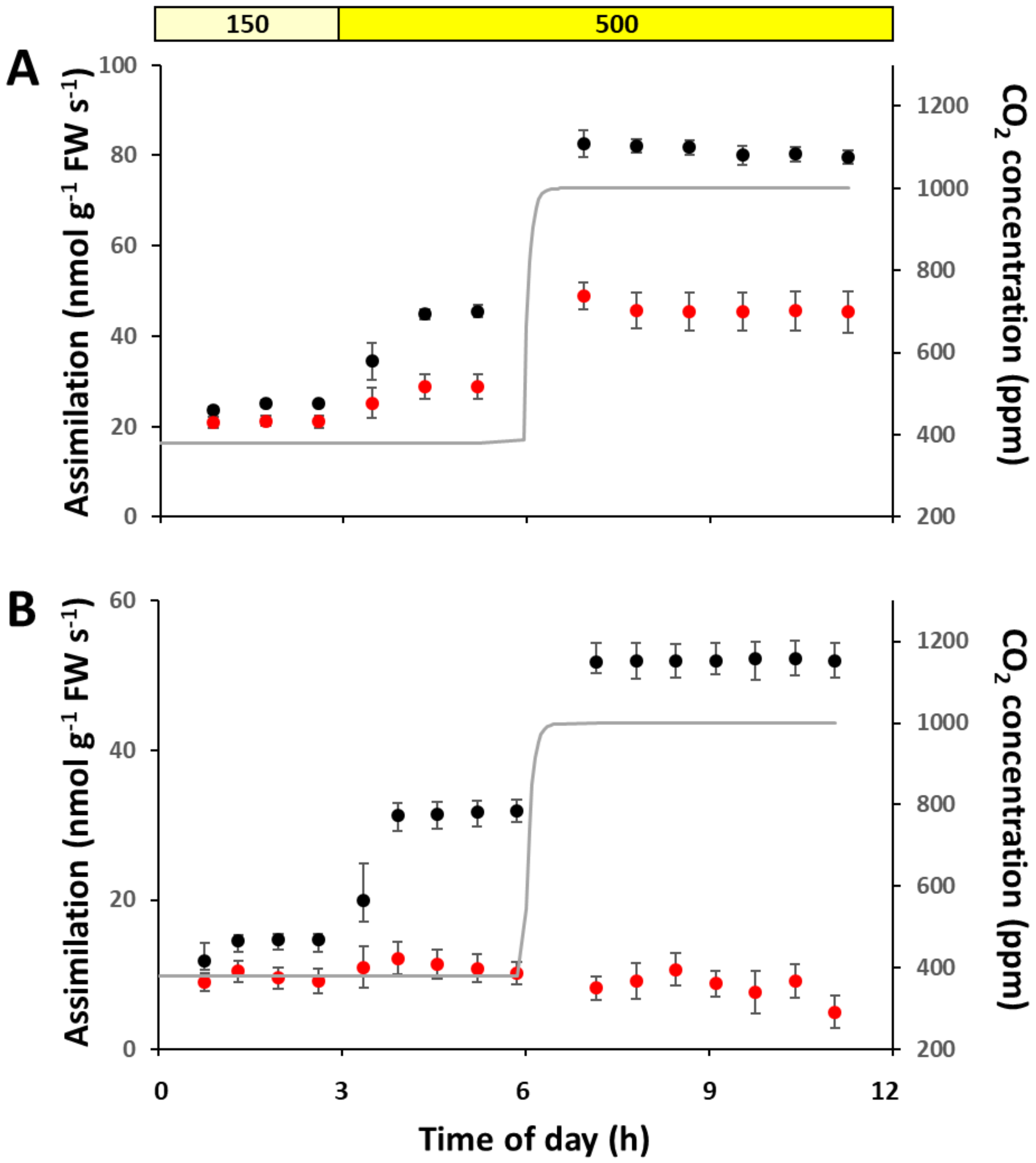

Supplementary Figure S3. Changes in photosynthetic rate in *fba2, fba3* and their respective wild types in response to elevated light and CO_2. Daytime photosynthetic carbon assimilation of the aerial parts of A) 28-day old *fba2*-1 (red) and Col-0 wild-type (black) plants and B) 50-day old *fba3* plants (red) and 28-day old Ler-0 wild-type (black) plants, measured by infrared gas analysis. The light intensity is indicated above the graphs. The grey line indicates the supplied CO_2 concentration, which steps from 380 to 1000 ppm. Mean values (± SE) from 4 (A) or 3 (B) biological replicates are given. Readings were paused during adjustment to the higher CO_2 concentration.

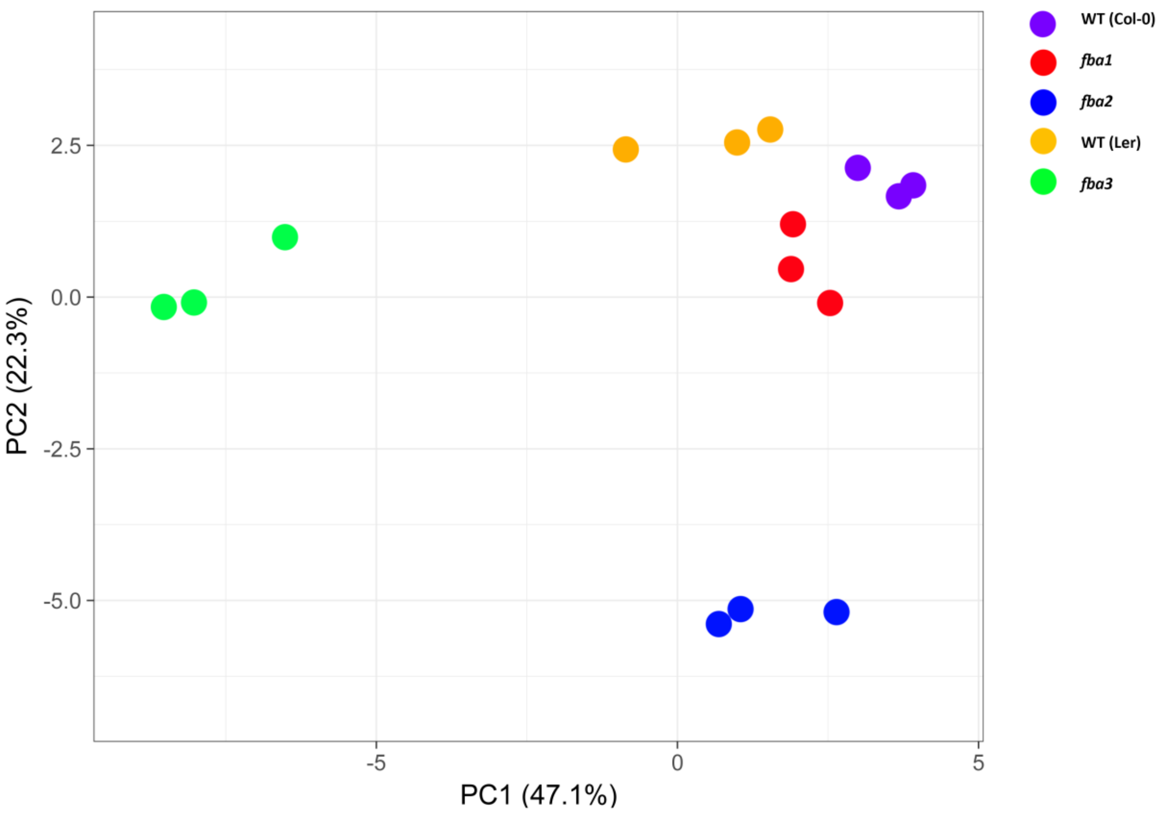

Supplementary Figure S4. PCA analysis of the shoot metabolomic measurements of the *fba1*-1, *fba2*-1 and *fba3*-1 mutants with the respective wild types. The analysis was carried out with ClustVis (Metsalu and Vilo, 2015).

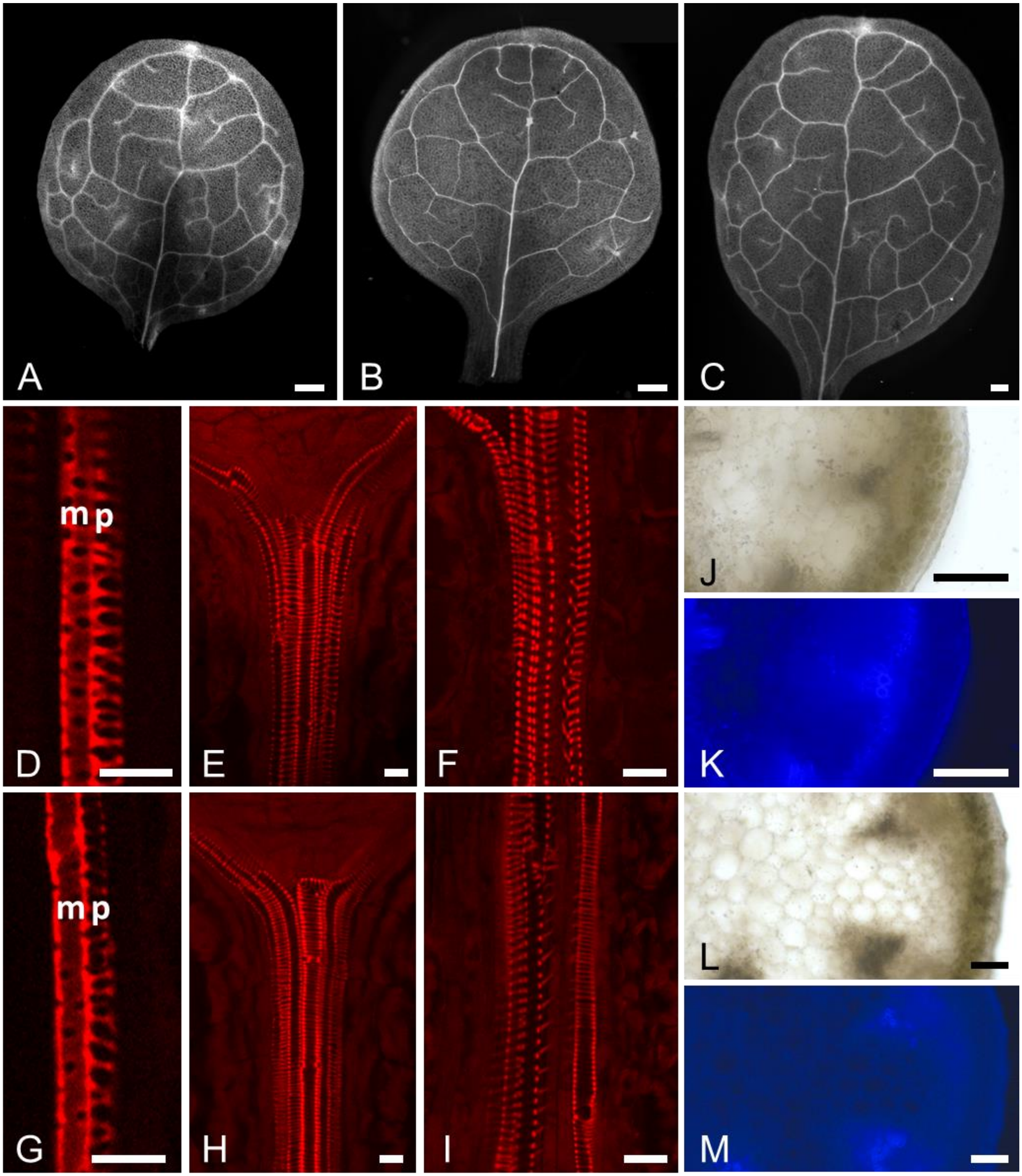
A
B
C
D
mp
E
F
J
K
G
mp
H
I
L
M

Supplementary Figure S5: Comparison of vascular patterns and xylem lignification in *fba3*-1 and its corresponding wild type (L*er*).

(A - C) Pattern of the vasculature in 1st true leaves of (A) *fba 3*-1 and (B, C) the wild type. Leaves in (A) and (B) were harvested from plants of approximately the same size, when *fba3*-1 plants were 2 months old and L*er* plants were 2 weeks old. The leaf in (C) is harvested from an 18 day-old L*er* plant, which had expanded further (note the different scale bar). No difference in the vascular patterns of mutant and wild-type was detected. (D - I) Wall structure of xylem vessels in different plant organs of 7 day-old seedlings of *fba3*-1 (D - F) and L*er* (G - I). Lignin, visualized by Basic Fuchsin staining revealed similar reticulate cell wall structure of protoxylem (p) and pitted cell wall structure of metaxylem (m) vessels in roots of the wild type and *fba3*-1 (D, G). In hypocotyls (E, H) and leaf major veins (F, I) xylem cell wall structure was similar in *fba3*-1 and the wild type. Sections through first inflorescence stem of *fba3*-1 (J, K) and L*er* (L, M) were visualized by bright field microscopy (J and L) and for lignin autofluorescence (fluorescence microscopy using a DAPI filter set - blue colour; K and M). Images were taken with the same exposure times. The *fba3*-1 stem section displays more lignified cells than the control, but note that the overall stem anatomy differed, with *fba3*-1 stems having smaller diameters (evident from the different scale bars). Scale bars: 1 mm in (A - C), 10 µm in (D - I), and 100 µm in (J - M)
